# Attention integrated deep learning models for interpretable multi-class IoT intrusion detection using SHAP

**DOI:** 10.3389/frai.2026.1825655

**Published:** 2026-06-03

**Authors:** Ramakrishnan Raman, Rahul Kumar, Benson Edwin Raj, Vishwesh Akre

**Affiliations:** 1 Higher Colleges of Technology - Dubai Men's Campus, Dubai, United Arab Emirates; 2 The University of the South Pacific, Suva, Fiji; 3 Higher Colleges of Technology - Fujairah Women's Campus, Fujairah, United Arab Emirates

**Keywords:** Convolutional Neural Network, GRU, HCRL dataset, Internet of Things, intrusion detection, Kitsune dataset, LSTM

## Abstract

The rapid growth of the Internet of Things (IoT) has increased the size, complexity, and vulnerability of network traffic, making intrusion detection a critical factor of modern cybersecurity. Traditional intrusion-detection systems (IDSs) analyze handcrafted features and rules to detect emerging attack patterns. To address these limitations, deep learning frameworks, such as attention-enhanced one-dimensional (1D) Convolutional Neural Network (CNN), Long Short-Term Memory (LSTM), and Gated Recurrent Unit (GRU), are analyzed for accurate and efficient multi-class attack detection. The proposed attention-enhanced 1D convolutional layers extract discriminative spatial features while focusing on the most relevant patterns within the network flow. The LSTM and GRU architectures analyzed long-range temporal dependencies present in sequential traffic data, enabling robust identification of subtle anomalies. The model was evaluated on two datasets: Hacking and Countermeasure Research Lab (HCRL) and the Kitsune IoT datasets, which represent diverse real-world benign and malicious traffic conditions. Experimental results demonstrate that the attention-enhanced 1D CNN achieved the highest performance of 98 and 87% on the Kitsune and HCRL datasets, respectively. SHapley Additive exPlanations (SHAP) based interpretability analysis shows how individual features contribute to predictions, highlighting the most significant features driving intrusion-detection decisions. The results confirm that incorporating attention mechanisms significantly enhances the discriminative capability, enabling more reliable classification of complex IoT attack types. The proposed approach effectively addresses key challenges in IoT intrusion detection by combining spatial and temporal deep learning components for deployment in intelligent real-time IoT network security systems.

## 1 Introduction

Rigorous growth in communication media, high-performance computing, and massive storage units, etc., has been observed over the past decade. Traditional security methods often fail, and struggle to manage uncertainties in real-time environments (Alabdulatif et al., 2021). According to recent literature, machine learning (ML) opportunities in cybersecurity include Why, What, and How in which “Why” explains the purpose of using ML, including prediction, prevention, detection, response, and monitoring. “What” specifies the technical layer being secured, such as the network, endpoint, application, and user or process. “How” refers to the methods used to ensure security within that specific area (Liu et al., 2018). Types of attacks have been increasing over the years (Kuypers et al., 2016) where network intrusion-detection system (NIDS) is a commonly implemented security measure. NIDS is a device or software that monitors all traffic passing a strategic point for malicious activities. In the recent literature, many NIDS datasets have been proposed and analyzed, where a few of them are publicly available to the research community. A few datasets that are publicly available are the Network Security Laboratory-Knowledge Discovery in Databases (NSL-KDD) dataset (Gurung et al., 2019; Ding and Zhai, 2020), University of New South Wales Network Benchmark Dataset 2015 (UNSW-NB15) (Moustafa and Slay, 2015), Kitsune (Mirsky et al., 2018), Coburg Intrusion Detection Data Sets-001 (CIDDS-001) (Damasevicius et al., 2020), Knowledge Discovery and Data Mining Cup (KDD CUP) (Divekar et al., 2018; KDD Cup 1999 Dataset, 1999), and Lithuanian Academic and Research Network (LITNET) dataset (Verma and Ranga, 2018). The extensive adoption of Internet of Things (IoT) devices has made life easier but has also introduced new cybersecurity challenges. Hackers are increasingly turning IoT devices into targets. They exploit security flaws to execute a variety of attacks, including botnet malware attacks, distributed denial-of-service (DDoS) attacks, surveillance, reconnaissance, and man-in-the-middle (MITM) attacks. Innovative detection techniques are required because traditional security systems cannot keep up with the dynamic and varied nature of IoT environments. Message Queuing Telemetry Transport (MQTT), a lightweight messaging protocol, is particularly vulnerable to cyberattacks due to its widespread use and inherent security challenges. The key contributions of this proposed work are as follows:

- The proposed model introduces an attention-enhanced one-dimensional 1D Convolutional Neural Network (CNN) architecture integrating both channel and spatial attention mechanisms without conventional pooling operations. This work recalibrates channel-wise and temporal features, enabling the model to retain fine-grained patterns while selectively emphasizing intrusion-relevant features.
- The study incorporated Long Short-Term Memory (LSTM) and Gated Recurrent Unit (GRU) architectures to capture long-range temporal dependencies, identifying behavioral patterns with a deeper understanding of temporal dynamics in multi-class intrusion detection.
- Attention-enhanced 1D CNN, LSTM, and GRU models are compared within a unified experimental setup using the HCLR and Kitsune datasets, showing the superior spatial-temporal feature learning capability of the attention-enhanced 1D CNN over the recurrent architectures.
- SHapley Additive exPlanations (SHAP)-based interpretability with attention mechanisms provides dual-level explainability, allowing both model feature prioritization using attention and feature contribution, enhancing transparency and trust in intrusion detection.
- The proposed model is validated using two heterogeneous IoT datasets (HCRL and Kitsune), showing the robustness and generalizability of the attention-enhanced feature learning across different representations, packet-level and statistical features.

The article is organized as follows: Section 2 details the existing research on IoT network intrusion detection, along with the limitations in handling complex and multi-class intrusion patterns. Section 3 presents the detailed architecture of the proposed attention-enhanced 1D CNN, along with LSTM and GRU models, and a unified preprocessing pipeline for consistent performance evaluation. Section 4 details experimental setup, dataset preprocessing, feature transformation, model training, and evaluation metrics. This section also includes a performance comparison among attention-enhanced 1D CNN, LSTM, and GRU models using SHAP-based interpretability analysis. Section 5 concludes the proposed work by summarizing key findings, the strengths of the proposed architecture, and future research directions in intrusion detection for IoT environments.

## 2 Related work

Abuzir and Abu Khalil (2025) used the Kitsune Fuzzing dataset to compare Linear Discriminant Analysis (LDA), Logistic Regression (LR), and a hybrid CNN + GRU + LSTM model for network intrusion detection. Logistic Regression achieved 79% accuracy with good interpretability, while the hybrid model attained improved accuracy at the cost of higher computational resources. Medina-Arco et al. (2024) utilized the University of Granada 2016 (UGR'16) dataset to address the issue of mislabeled and contaminated network traffic data, which affects the performance of network intrusion-detection systems. A two-stage methodology was proposed to evaluate and detect hidden anomalies in the Kitsune dataset by selecting the optimal training subset to improve anomaly detection. Experimental results achieved an area under the curve (AUC) of up to 0.95, while hidden anomalies reduced performance to 0.89 for the Kitsune dataset.

Hirano and Kobayashi (2025) investigated ransomware attacks using a novel detection approach that leverages low-level storage, memory, and network traffic generated by the hypervisor to enhance defense-in-depth protection. Utilizing the lightweight Kitsune NIDS, they successfully detected the data exfiltration phase of ransomware attacks, improving the macro-F-score by 0.166 and enhancing detection performance against advanced ransomware threats. Doostali et al. (2025) explored the use of neural networks and machine learning models for NIDSs using the botnet malware attacks subset of the Kitsune dataset. To address the high computational cost of deep learning models, they introduced a feature selection method based on the Efficiency Detector Value (EDV), enabling more efficient offline analysis. Among the evaluated algorithms, deep learning, linear regression, Random Forest (RF), and decision tree classifiers, the decision tree achieved the best performance with a 99.97% recall, showing a 0.28% improvement over deep federated learning and a 67% reduction in runtime.

Dina and Manivannan (2021) presented a comprehensive survey of ML-based intrusion-detection systems (IDSs) developed over the past decade. Their review categorized existing approaches into signature and anomaly detection, highlighting that signature-based systems identify known attack patterns, whereas anomaly based systems can detect unknown or emerging threats by modeling user behavior. Qazi et al. (2023) proposed a hybrid deep-learning-based network intrusion-detection system (HDLNIDS) that integrates Convolutional Neural Networks and Recurrent Neural Networks (RNNs) for effective network attack detection. CNN captured local spatial features while the RNN extracted sequential dependencies to enhance detection accuracy. Canadian Institute for Cybersecurity Intrusion-Detection System (CICIDS)-2018 achieved an accuracy of 98.90%, outperforming various intrusion detection approaches.

Kumar et al. (2022) enhanced Internet of Things security by developing Network-Based Intrusion Prevention Systems (NBIPSs) to protect IoT and cloud network infrastructures from unauthorized access. The proposed NBIPS inspects network activity streams to detect and block malicious behavior using IoT signature protocols. This system employs inline sensors for real-time monitoring and prevention, providing an additional security layer that effectively identifies and mitigates network misuse and intrusion attempts in IoT environments. Zeghida et al. (2025) proposed a unified intrusion-detection system (UIDS) designed for IoT environments to safeguard networks from four major attack types, such as exploits, denial-of-service (DoS), probes, and generic attacks, as well as to identify normal traffic. The proposed system utilized the UNSW-NB15 dataset to improve the detection of modern attack patterns. Zeghida et al. (2025) conducted a study using the MQTT-IoT-IDS2020 dataset to classify network traffic into normal and multiple attack types, including brute force, scan A, scan sU, and Sparta. Five classifiers, like Random Forest (RF), linear RBF SVM, CNN, and CNN + LSTM, were evaluated, where the RF classifier achieved the highest accuracy. The study also introduced XMID + MQTT, an explainable intrusion-detection framework that employs SHAP and LIME to interpret model predictions. This approach enhanced the transparency and interpretability of artificial intelligence (AI)-driven IDS, making them more trustworthy and effective for securing IoT environments.

Jablaoui et al. (2025) proposed a hybrid deep learning model that integrates CNN and GRU to capture both spatial and temporal features of network traffic data. The model was evaluated on two different datasets for classification tasks. Experimental results demonstrated that the CNN + GRU hybrid model achieved better performance with 98.60% accuracy on the NetFlow version of NetFlow-based University of New South Wales—Network Behavior 15 (NF-UNSW-NB15) and 97.95% accuracy on the NetFlow version of Communications Security Establishment—CICIDS-2018 (NF-CSE-CICIDS-2018), outperforming standalone models and existing approaches in IoT intrusion detection. Rahma Jablaoui and Noureddine Liouane (Jablaoui and Liouane, 2025) proposed an advanced deep learning model for an intrusion-detection system combining Convolutional Neural Networks with four Recurrent Neural Network variants like LSTM, Bidirectional Long Short-Term Memory (BiLSTM), GRU, and Bidirectional Gated Recurrent Unit (BiGRU) to extract both spatial and temporal features from IoT network traffic. The models were evaluated on the NetFlow University of Queensland Network Intrusion-Detection System (NF-UQ-NIDS) dataset, which integrates multiple NetFlow datasets. Experimental results demonstrated better performance with the CNN-BiGRU model, achieving the highest accuracy of 98.69% on NetFlow-based Botnet Internet of Things (NF-BoT-IoT) and 98.62% on NF-UNSW-NB15, while the CNN + GRU model reached 97.97% accuracy on NF-CSE-CICIDS-2018. These results confirm the effectiveness of combining CNNs with RNN variants in enhancing intrusion-detection accuracy and reducing false alarm rates (FARs) for IoT network security. Shahad Altamimi and Qasem Abu Al-Haija (Altamimi and Abu Al-Haija, 2024) proposed an extreme learning machine-based intrusion-detection system (ELM-IDS) to enhance the performance and adaptability of IDSs in IoT communication networks. The model was evaluated using two benchmark datasets, NSL-KDD (Tavallaee et al., 2009) and Distilled-Kitsune (Abu Al-Haija and Al-Badawi, 2022), to assess its ability to handle high-dimensional, unbalanced data. Experimental results demonstrated outstanding performance, achieving better accuracy in binary and multi-class classification. The study highlighted the efficiency, flexibility, and scalability of the ELM-IDS approach as a promising solution for next-generation IoT network security.

Hazman et al. (2023) developed IDS-Smart Environments using Ensemble Learning (SIoEL), an ensemble-learning-based intrusion-detection framework for IoT-enabled smart city environments. The model integrates Adaptive Boosting (AdaBoost) with multiple feature selection techniques to optimize anomaly detection. The model was evaluated across different datasets, achieving better accuracy with a detection time of 0.02 s, outperforming existing IDS methods in both detection precision and computational efficiency. Maray et al. (2022) introduced a Harmony Search Algorithm-based Feature Selection with Optimal Convolutional Autoencoder (HSAFS + OCAE) model for intrusion detection in Software Defined Networking (SDN)-enabled IoT environments. The proposed framework employed Harmony Search Algorithm (HSAFS) for optimal feature selection, followed by a Convolutional Autoencoder (CAE) for intrusion classification, and utilizes the Artificial Fish Swarm Algorithm (AFSA) for hyperparameter fine-tuning. Experimental evaluation demonstrated that HSAFS+OCAE achieved the highest classification performance, with an accuracy of 93.44%, outperforming existing models such as LSTM-CNN, Bi-LSTM, and Cu-DNNGRU + Cu-BLSTM, demonstrating its effectiveness in detecting intrusions in high-traffic IoT networks.

Xue et al. (2025) proposed a Hybrid Autoencoder + Hybrid ResNet + LSTM model that combined CNN- and GRU-based autoencoders for dimensionality reduction and feature extraction. The model is evaluated on three benchmark datasets, such as UNSW-NB15, NSL-KDD, and CICIDS-2018, attaining detection accuracies of 95.7, 94.9, and 96.7%, respectively, outperforming several existing IDS models and demonstrating superior adaptability across diverse network environments. Li et al. (2024) introduced a hybrid detection architecture combining a Variational Auto-Encoder (VAE) and Generative Adversarial Networks (GAN) to address class imbalance in anomalous network traffic. The proposed VAE-Wasserstein Generative Adversarial Network (WGAN) model generated balanced training data, followed by a hybrid detection framework using stacked LSTM and Multi-Scale (MS) CNNs for feature extraction and fusion. Experimental results showed better performance, achieving 83.45% accuracy and 83.69% F1-score on the NSL-KDD dataset and 98% accuracy on the Aegean Wi-Fi Intrusion Dataset (AWID), outperforming several existing intrusion-detection models.

Vibhute et al. (2024) evaluated multiple models, such as Support Vector Machine (SVM), Random Forest (RF), eXtreme Gradient Boosting (XGBoost), Neural Network, and Keras, on the KDD and NSL-KDD datasets during the training phase. The results showed that RF achieved accuracies of 98.99% on the KDD dataset and 97.93% on the NSL-KDD dataset, with the lowest false alarm rates (FARs). These findings indicate that RF provides the most reliable detection capability with efficient testing time, making it highly effective for intrusion detection in the proposed ANIDINR system.

Faiaz et al. (2024) proposed a novel approach to convert tabular network intrusion data into image form, making it compatible with Convolutional Neural Networks. Using the NSL-KDD dataset, each data row was transformed into an image using the Hue-Saturation-Value (HSV) and Viridis colormaps. Five CNN architectures were tested using transfer learning, with ResNet18 achieving the best accuracy of 98.91% and an F1-score of 0.91. Igor Zelichenok and Kotenko (2024) developed a network intrusion-detection system that integrates Big Data with machine learning models featuring LSTM layers to detect complex, multi-stage network attacks efficiently. The system was evaluated using the Kitsune dataset for a binary classification task. Experimental results demonstrated better performance, achieving an accuracy of 93% with a loss of 0.03, highlighting the capability to analyze large-scale network traffic and effectively identify intrusions in real time. (Abu Al-Haija and Al-Badawi 2022) developed and evaluated ML-based network intrusion-detection systems for IoT networks, emphasizing lightweight, anomaly based detection approaches. Six supervised learning methods in three categories, like ensemble methods, Neural Networks, and kernel methods, were compared using the distilled Kitsune (Mirsky et al., 2018) and NSL-KDD datasets. Experimental results showed that the Ensemble Boosted Trees (EBT) model achieved the highest accuracy of 99.20%, outperforming other state-of-the-art systems such as XGBoost (97.81%) and Spatial-Convolutional Neural Network (S-CNN) (98.20%). The study concluded that ensemble models deliver higher accuracy and lower error rates while Neural Networks provide faster inference, making them suitable for high-bandwidth IoT environments. Table 1 presents details of the comparison among various research studies in the literature.

**Table 1:** Comparison of the related works done in intrusion detection.

References	Model/method	Dataset(s)	Performance	Inference
Abuzir and Abu Khalil (2025)	LDA LR CNN–GRU–LSTM hybrid	Kitsune Fuzzing dataset	Accuracy: 79%	High computation cost
Medina-Arco et al. (2024)	Two-stage learning model	UGR'16, Kitsune NIDS	AUC: 0.95	Performance dropped to 0.89 under contamination
Hirano and Kobayashi (2025)	Hypervisor-based detection using low-level system features	Kitsune NIDS	Macro-F-score improvement: +0.166	Detected ransomware data exfiltration
Doostali et al. (2025)	Feature selection via EDV and DT	Kitsune (botnet malware subset)	Decision tree: 99.97% recall	0.28% better than deep federated learning
Qazi et al. (2023)	HDLNIDS (Hybrid CNN + RNN)	CICIDS-2018	Accuracy: 98.90%	Outperformed existing IDSs
Zeghida et al. (2025)	Unified intrusion-detection system	UNSW-NB15	Improved accuracy over Ensemble Network Anomaly Detection System (ENADS) and DENDRON - A Genetic Trees Driven Rule Induction for Network Intrusion Detection Systems	Detected 4 attack types along with normal
Jablaoui et al. (2025)	CNN–GRU hybrid	NF-UNSW-NB15 NF-CSE-CICIDS-2018	98.60% 97.95%	Outperformed standalone models
Jablaoui and Liouane (2025)	CNN + RNN variants	NF-UQ-NIDS	CNN-BiGRU: 98.69% CNN-GRU: 97.97%	Better accuracy
Altamimi and Abu Al-Haija (2024)	Extreme learning machine IDS (ELM-IDS)	NSL-KDD Distilled-Kitsune	Better accuracy for binary and multi-class	High efficiency and scalability
Hazman et al. (2023)	IDS-SIoEL (AdaBoost + feature selection)	IoT-23 BoT-IoT Edge-IIoT	Accuracy: ~99.9%	33.68s learning time; 0.02156s detection time
Maray et al. (2022)	HSAFS-OCAE	SDN enabled IoT	Accuracy: 93.44%	Outperformed LSTM-CNN, Bi-LSTM, Cu-DNNGRU, Cu-BLSTM
Xue et al. (2025)	Hybrid Autoencoder + Hybrid ResNet-LSTM	NSL-KDD UNSW-NB15 CICIDS-2018	95.7% 94.9% 96.7%	Effective across different datasets
Li et al. (2024)	VAE-WGAN + LSTM + MSCNN	NSL-KDD, AWID	83.45% accuracy (NSL-KDD), 98.9% (AWID);	Solved class imbalance issue
Vibhute et al. (2024)	SVM, RF, XGBoost, Neural Network, Keras	KDD NSL-KDD	RF: 98.99% 97.93%	Lowest false alarm rate
Faiaz et al. (2024)	CNN	NSL-KDD	Accuracy: 98.91% F1-score: 0.91	Novel tabular-to-image mapping
Zelichenok and Kotenko (2024)	Big Data + LSTM-based NIDS	Kitsune dataset	Accuracy: 93%	Loss 0.03; efficient real-time intrusion detection
Abu Al-Haija and Al-Badawi (2022)	Ensemble methods, Neural Networks, Kernel methods	NSL-KDD	EBT: 99.20% XGBoost: 97.81% S-CNN : 98.20%	Best for IoT NIDS

Wang et al. (2026) proposed a Knowledge Graph Large Language Model **(**KG-LLM) framework that integrated knowledge graphs with large language models to enhance intrusion detection in sixth-generation (6G)-enabled Industrial Internet of Things (IIoT) environments, thereby improving the accuracy and robustness through knowledge and reasoning over complex spatial–temporal data. Saidi (2026) proposed NIDS-β^*^, an LLM-inspired intrusion-detection framework integrating transformer embeddings and statistical features, achieving 98.6% accuracy on CICIDS-2018 and 97.8% on UNSW-NB15. Saidi (2025) proposed IDS-GPT, a ChatGPT-based intrusion-detection framework that achieves high accuracy and low false-positive rates on the CICIDS-2018 dataset, outperforming models such as decision trees, CNN-BiLSTM, and GBM. Praveen et al. (2026) proposed a CNN Stacked LSTM with multi-head attention, attaining 99.99% accuracy on the UNSW-NB15 dataset by effectively capturing spatial–temporal features for intrusion detection. Ishtiaq et al. (2025) developed CST-AFNet, a dual-attention framework that combines Multi-Scale CNN, BiGRU, and channel-temporal attention to achieve highly accurate, scalable intrusion detection in IoT networks. B AlOmar et al. (2023) proposed attention-enhanced LSTM and BiLSTM models for intrusion detection, achieving a 93% detection rate on the UNSW-NB15 dataset by leveraging temporal dependencies and important features. Chen et al. (2026) proposed a GBIFS, an intrusion-detection framework with an improved distance metric with intuitionistic fuzzy sets, attaining high accuracy and robust performance in various IIoT datasets. Almseidin (2026) proposed a hybrid IDS with Fuzzy Rule Interpolation and Neural Networks, using a fusion mechanism to achieve robust, adaptable intrusion detection across various benchmark datasets with improved generalization and reduced false positives. Almasalmeh and Trabelsi (2019) enhanced the Dendritic Cell Algorithm, inspired by the human immune system, to effectively detect malicious TCP port scanning and DoS attacks with improved detection efficiency.

## 3 Proposed work

This section details the model's evaluated to analyze the several types of network attacks in an IoT environment. Attention-enhanced 1D CNN, LSTM, and GRU deep learning frameworks are analyzed for sequential data classification. The attention-enhanced 1D CNN model captures local temporal patterns through convolutional filters and incorporated residual connections, along with channel- and spatial-attention-enhanced feature representations on the most informative time steps. The LSTM model extracted long-term dependencies from sequential data, and the integrated attention mechanisms focused on the most significant temporal features to improve classification accuracy. GRU model efficiently identified temporal dependencies with a simpler gating mechanism compared to LSTM and emphasized crucial sequence features by the attention layer. Dropout regularization is applied in all models to prevent overfitting and ensure robust learning.

### 3.1 Attention-enhanced 1D CNN

The proposed attention-enhanced 1D CNN architecture analyzes the input using a 1D convolutional layer with 64 filters of kernel size 3 and the same padding, with the Rectified Linear Unit (ReLU) activation function. This layer extracts the initial local spatial–temporal patterns from the pre-processed IoT network intrusion-detection data. A dropout layer of 20% is introduced to reduce the tendency of neurons to rely on each other and to improve the model's generalization. This is followed by another Conv1D layer with 64 filters and a kernel size of 3, which further strengthens the learning of low-level feature representations. The convolution operation for the input data $X_{t}\;\epsilon \;R^{Lx1}$ is given in Equation 1.


$$\begin{array}{l} F_{t}^{l}=ReLU\;\left(W^{l}\;*\;X_{t}+\;b^{l}\right) \end{array}$$ (1)

where *W*^*l*^ denotes the convolution kernel at layer *l* and ^*^ represents 1D convolution. A dropout layer is applied to regularize the feature maps. After these initial convolution layers, the feature map is refined using two attention modules to enhance discriminative features. The channel attention block (Woo et al., 2018) learns which channels are most important by using both global average pooling (*F*_*avg*_) and max pooling (*F*_max_). These features are processed by a shared Multi-Layer Perceptron (MLP) consisting of two fully connected layers to generate an intermediate attention feature map: *W*_*avg*_ and *W*_max_. The outputs of these two branches are added and processed by a sigmoid activation to generate the final channel attention features $\left(M_{c}^{F}\right)$ to highlight the channels that include more precise intrusion-detection-related features while reducing the influence of less important features, as shown in Equation 2. This attention map is then multiplied element-wise with the original feature map to enhance the channels that include highly discriminative intrusion-related information while suppressing those with weaker relevance.


$$\begin{array}{l} M_{c}^{F}=\;\sigma \;\left(W_{1}\left(W_{0}\left(F_{avg}\right)\right)+W_{1}\left(W_{0}\left(F_{\max}\right)\right)\right) \end{array}$$ (2)

Spatial attention block refines the feature map along the temporal dimension by identifying which time steps contribute to intrusion-associated patterns. The module initially computes two temporal descriptors, such as the average-pooled map *F*_*avg*_ and a maximum-pooled map *F*_max_. The two maps are then concatenated and processed by a convolutional layer with a kernel size of 7, enabling the model to learn wider contextual dependencies. The resulting output is transformed through a sigmoid function to generate the spatial attention mask (*M*_*s*_) as shown in Equation 3.


$$\begin{array}{l} M_{s}=\;\sigma \left(M_{s}\;*\;\left[F_{avg};\;F_{\max}\right]\right) \end{array}$$ (3)

This attention mask is applied element-wise to the channel-refined feature map *F*_*c*_ generating the spatially enhanced feature set as shown in Equation 4.


$$\begin{array}{l} F_{s}=\;M_{s}\;⊙\;F_{c} \end{array}$$ (4)

The proposed architecture highlights the most informative temporal positions associated with malicious activities while removing the influence of irrelevant or noisy regions in the data. Following the attention refinement process, the model further processes the features with a Conv1D layer with 32 filters and a dropout layer to extract more detailed temporal patterns and control overfitting. These features are processed through another Conv1D layer with 32 filters and a dropout layer to progressively reduce feature complexity and stabilize learning. The resulting feature map is then flattened and passed into a dense layer with 128 neurons using ReLU activation, allowing the model to capture higher level abstractions of intrusion behavior as shown in Equation 5.


$$\begin{array}{l} h=ReLU\left(W_{h}z+\;b_{h}\right) \end{array}$$ (5)

A 30% dropout rate is applied to regularize dense features and improve generalization. SoftMax (*o*) generates the probability distribution across the intrusion classification, indicating the model's predicted class for *k* intrusion classes as shown in Equation 6.


$$\begin{array}{l} y_{k}=\;\frac{e^{o_{k}}}{\sum_{j=1}^{k}e^{o_{j}}} \end{array}$$ (6)

Where *o* is defined as given in Equation 7, where *W*_*o*_ is the weights of the output layer, *b*_*o*_ is the bias vector of the output layer and *h* is the input to the output layer.


$$\begin{array}{l} o=\;W_{o}h+\;b_{o} \end{array}$$ (7)

**Figure 1:**
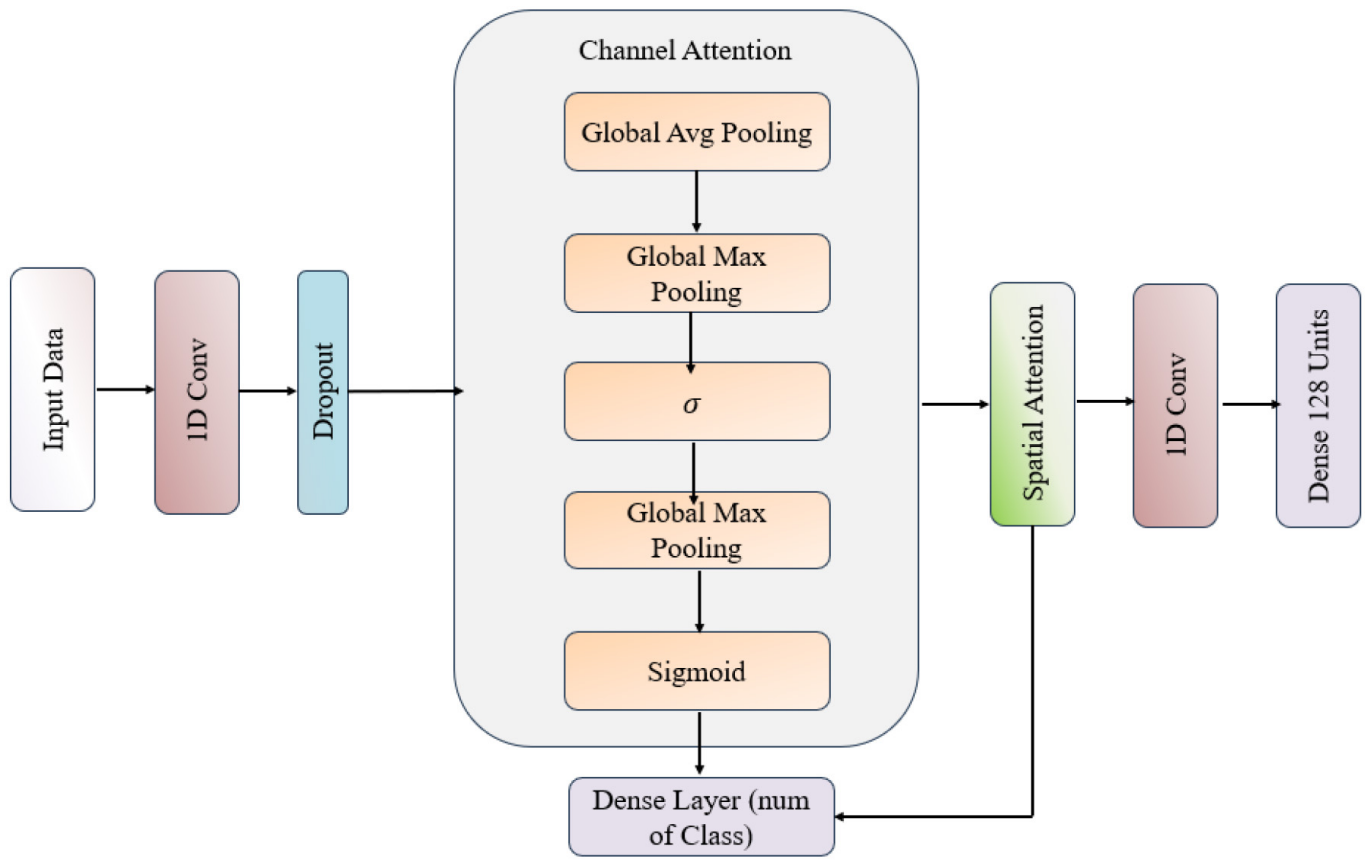
Architecture of the attention-enhanced 1D CNN.

Unlike traditional CNN architectures, this model avoids pooling layers such as max or average pooling. Pooling layers tend to compress the temporal dimension and may eliminate fine-grained variations that are often critical for intrusion detection in IoT environments. Instead of using a fixed down-sampling channel, spatial attention mechanisms are incorporated to serve as adaptive and learnable selectors of informative features. These attention modules enhanced relevant patterns without removing temporal features. The Conv1D layers progressively expand the receptive field and extract hierarchical features, while dropout provides regularization, eliminating the need for dimensionality reduction through pooling operations. This design, shown in Figure 1 retains temporal fidelity and maintains high sensitivity to small yet meaningful anomalies in network traffic, making it well-suited for real-time IoT intrusion detection.

### 3.2 Long Short-Term Memory

Long Short-Term Memory (Hochreiter and Schmidhuber, 1997) is an improved version of the RNN to efficiently capture the long-term dependencies in data. It addresses gradient commonly encountered in traditional RNNs. LSTM is widely used in applications due to its ability to selectively remember or forget information over time. LSTM included an additional memory cell that carries long-term information across time steps. The flow of information within the memory cell is controlled by three gates: the forget gate, input gate, and output gate. These gates determine what information to store, update, or discard, allowing the network to retain relevant features for longer periods.

#### 3.2.1 Forget gate

The forget gate adopts the information from the preceding cell state, which should be rejected. It takes the current input *x*_*t*_ and the previous hidden state *h*_*t*−1_ multiplies them with the respective weights, adds a bias, and processes through a sigmoid activation function as shown in Equation 8.


$$f_{t}=\;\sigma (W_{f}\;\cdot \;[h_{t-1,}\;x_{t}]+\;b_{f}$$ (8)

where *f*_*t*_ represents the forget gate activation vector, where the values range between 0 and 1, *W*_*f*_ refers to the weight of the forget gate, *b*_*f*_ refers to the bias term for the forget gate, σ represents the sigmoid activation function, [*h*_*t*−1_, *x*_*t*_] refers to the concatenation of the previous hidden state and current input.

#### 3.2.2 Input gate

A sigmoid layer chooses which must be updated, and the *tanh* creates new candidate values for the cell state as given in Equations 9, 10.


$$i_{t}=\;\sigma \;(W_{i}\;\cdot [h_{t-1,}\;x_{t}]+\;b_{f}$$ (9)

$${\hat{C}}_{t}=\;tanh\;(W_{c}\;\cdot [h_{t-1,}\;x_{t}]+\;b_{c}$$ (10)

Then the new cell state is updated as given in Equation 11.


$$\begin{array}{l} C_{t}=\;f_{t\;}\;⊙\;C_{t-1}+\;i_{t\;}\;⊙{\hat{\;C}}_{t} \end{array}$$ (11)

Where *i*_*t*_ represents the input gate activation vector, ${\hat{C}}_{t}$ shows the candidate cell state, *C*_*t*−1_ denotes the previous cell state and ⊙ shows the element-wise multiplication.

#### 3.2.3 Output gate

The output gate decides which part of the current cell state should be sent as the hidden state *h*_*t*−1_ which acts as the output for the current time step. The gate activation is computed as given in Equation 12.


$$o_{t}=\;\sigma \;(W_{o}\;\cdot [h_{t-1,}\;x_{t}]+\;b_{o}$$ (12)

and the hidden output state is obtained by applying a *tanh* activation to the cell state and multiplying it element-wise with the output gate activation as shown in Equation 13.


$$\begin{array}{l} h_{t}=o_{t}\;⊙\;tanh\left(C_{t}\right) \end{array}$$ (13)

**Figure 2:**
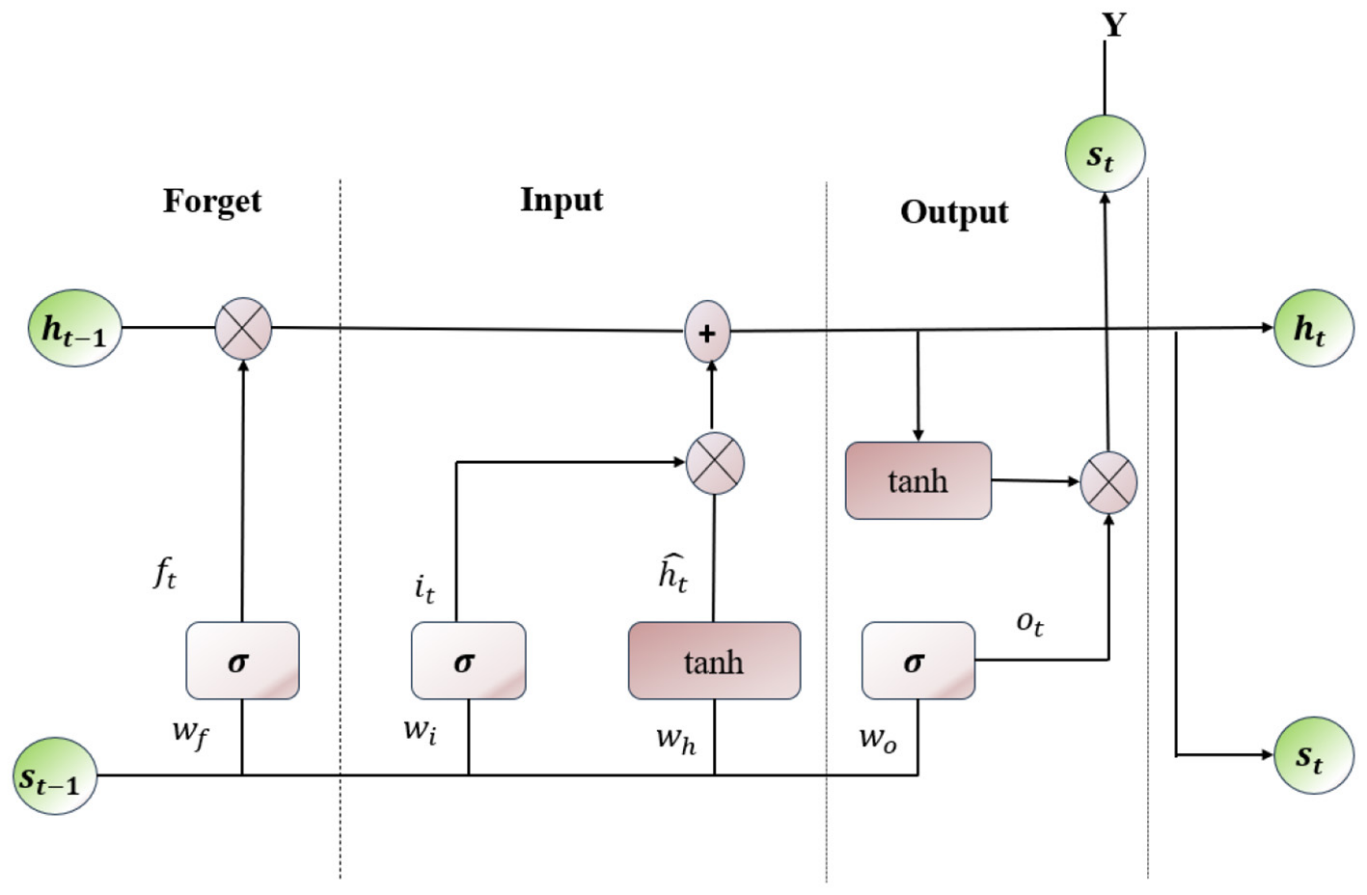
Overall flow of the LSTM architecture.

Where *o*_*t*_ refers to the output gate activation vector, *h*_*t*_ refers to the current hidden output state and *C*_*t*_ current cell state. This model captures long-term dependencies, effectively addresses vanishing gradient problems, and enables selective information retention via gating mechanisms, and is suitable for sequential and temporal tasks. This enables them to model complex temporal relationships and dependencies across long sequences efficiently. The overall flow of the LSTM model is shown in Figure 2.

#### 3.2.4 Gated recurrent unit

**Figure 3:**
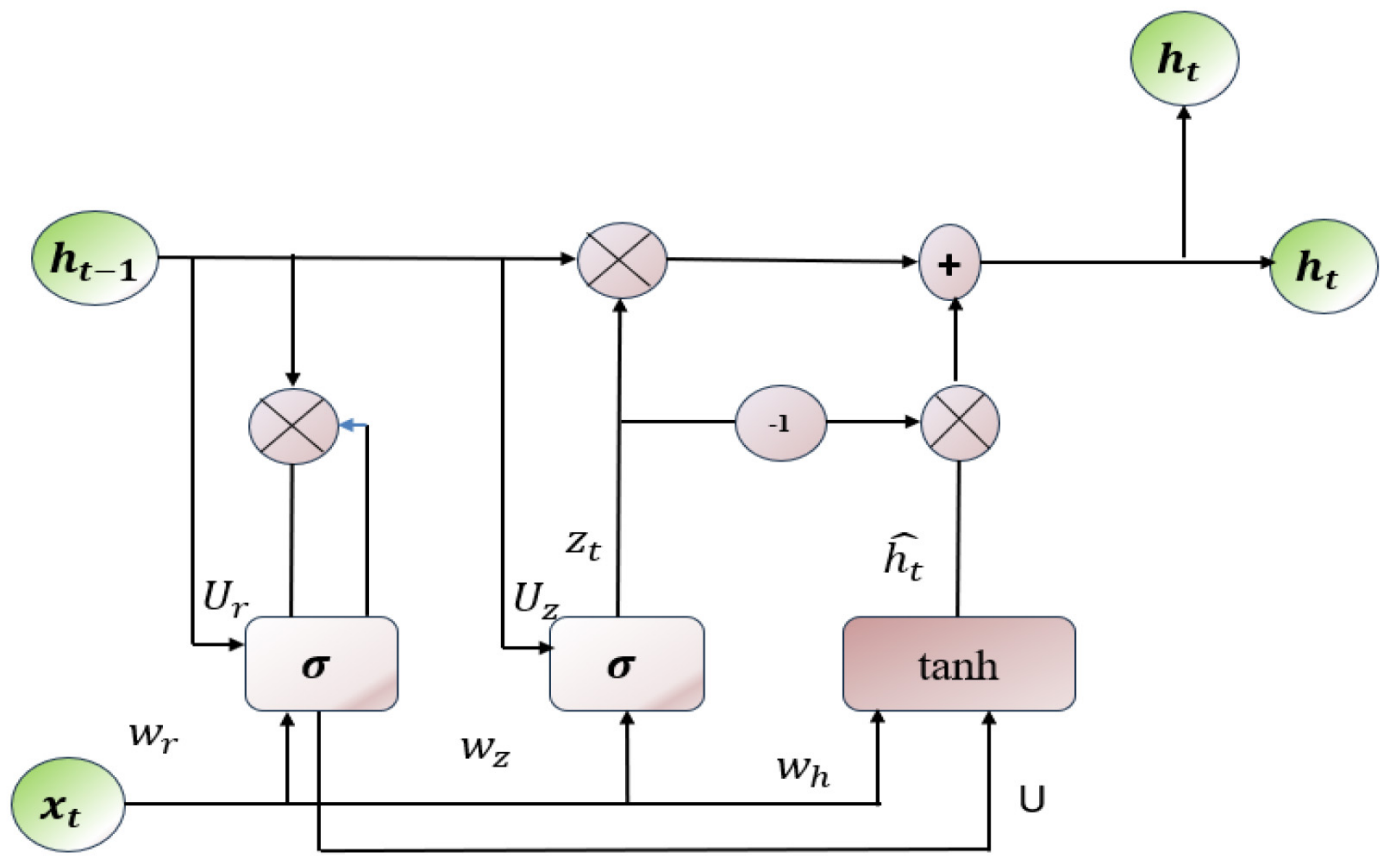
Overall flow of the GRU architecture.

GRU introduced by Chung et al. (2014) is a simplified variant of RNN designed to efficiently identify temporal dependencies as shown in Figure 3. The gating mechanisms control the flow of information and determine which parts of the past should be remembered and which should be forgotten. GRUs achieve similar performance to LSTMs but with a simpler architecture and fewer parameters. GRUs merge the cell state and hidden state into a single vector and use only two main gates. Update Gate (*z*_*t*_) regulates which value from the previous hidden state must be passed forward to the next time step, and the Reset Gate (*r*_*t*_) controls the value from the previous state to be forgotten. The reset gate determine how much of the previous hidden state to ignore, as given by Equation 14.


$$r_{t}=\;\sigma \;(W_{r}\;\cdot [h_{t-1,}\;x_{t}]$$ (14)

where *r*_*t*_ is the reset gate vector, *W*_*r*_ is the weight of the reset gate, *h*_*t*−1_ is the previous hidden state, *x*_*t*_ is the current input and σ denotes the sigmoid activation function. The update gate selects the new information to add to the hidden state, as given in Equation 15.


$$z_{t}=\;\sigma \;(W_{z}\;\cdot [h_{t-1,}\;x_{t}]$$ (15)

where *z*_*t*_ is the update gate vector and *W*_*z*_ is the weight matrix for the update gate. The candidate hidden state $\hat{h_{t}}\;$is computed using the reset gate as shown in Equation 16.


$$\begin{array}{l} \hat{h_{t}}=tanh\left(W_{h\;}\;\cdot \left[\;r_{t}\;⊙\;h_{t-1}+\;x_{t\;}\right]\right) \end{array}$$ (16)

where $\hat{h_{t}}$ is the potential new hidden state, *W*_*h*_ is the weight of the candidate hidden state and ⊙ denotes element-wise multiplication. The hidden state is controlled by the update gate as given by Equation 17, where *h*_*t*_ is the final hidden state for the current time step.


$$\begin{array}{l} h_{t}=\left(1-\;z_{t}\;\right)⊙\;h_{t-1}+z_{t}\;⊙\;\hat{h_{t}} \end{array}$$ (17)

## 4 Results and discussion

This section details the dataset used for analysis, preprocessing, attention-enhanced 1D CNN, LSTM, and GRU models, training, validation, and testing procedures, along with hyperparameter tuning for intrusion-detection classification.

### 4.1 Experimental setup

The proposed attention-enhanced 1D CNN, LSTM, and GRU were implemented and evaluated on a 64-bit Windows 10 operating system. Table 2 presents the configuration used for training and testing. The model development and experimentation were carried out using Python and deep learning frameworks, and the intrusion-detection dataset was obtained from Kaggle.

**Table 2:** Computer configuration of the proposed model.

Item	Configuration
Processor	11th Gen Intel^®^ Core™ i7-1165G7 @ 2.80 GHz
Graphics card	NVIDIA GeForce MX330 + Intel Iris Xe Graphics (Integrated)
Random access memory (RAM) size	8 GB
Hard-disk size	2 TB
Cores	4 cores, 8 threads

### 4.2 Dataset description

The proposed study analyses two intrusion and detection datasets. The dataset description is detailed in the following section.

#### 4.2.1 Hacking and Countermeasure Research Lab dataset

**Figure 4:**
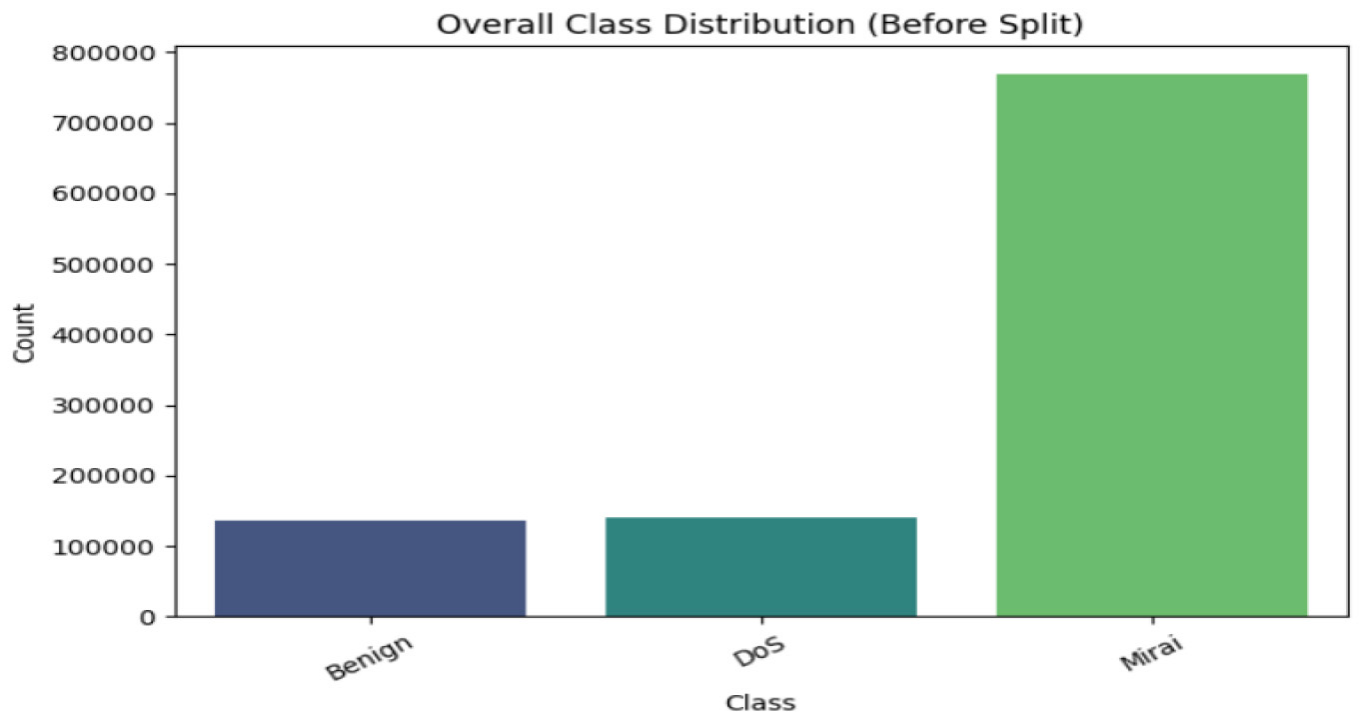
Overall class distribution of the HCRL data.

The IoT Environment Dataset developed by the Hacking and Countermeasure Research Lab (HCRL) (Han et al., 2018) was analyzed in this proposed work. It includes network traffic data collected from IoT devices, such as the South Korea's first AI-powered virtual assistant, developed by SK Telecom (SKT NUGU) smart speaker and EZVIZ Wi-Fi camera, operating on a shared wireless network. The dataset consists of approximately 2.9 million packets, including 1,756,276 normal, 64,646 DoS, and 1,038,000 Mirai botnet packets, including various attacks like Synchronize (SYN) flooding, User Datagram Protocol (UDP) flooding, Acknowledge (ACK) flooding, and Hypertext Transfer Protocol (HTTP) flooding. The proposed work analyzed only three major classes, like normal, DoS, and Mirai. Each data included features such as packet length, protocol type, along with source and destination IP octets. The dataset was preprocessed to remove missing or redundant entries and standardized before trained with deep learning models. The Figure 4 shows the overall class distribution of the data included in the intrusion-detection dataset.

#### 4.2.2 Kitsune Network Attack dataset

**Figure 5:**
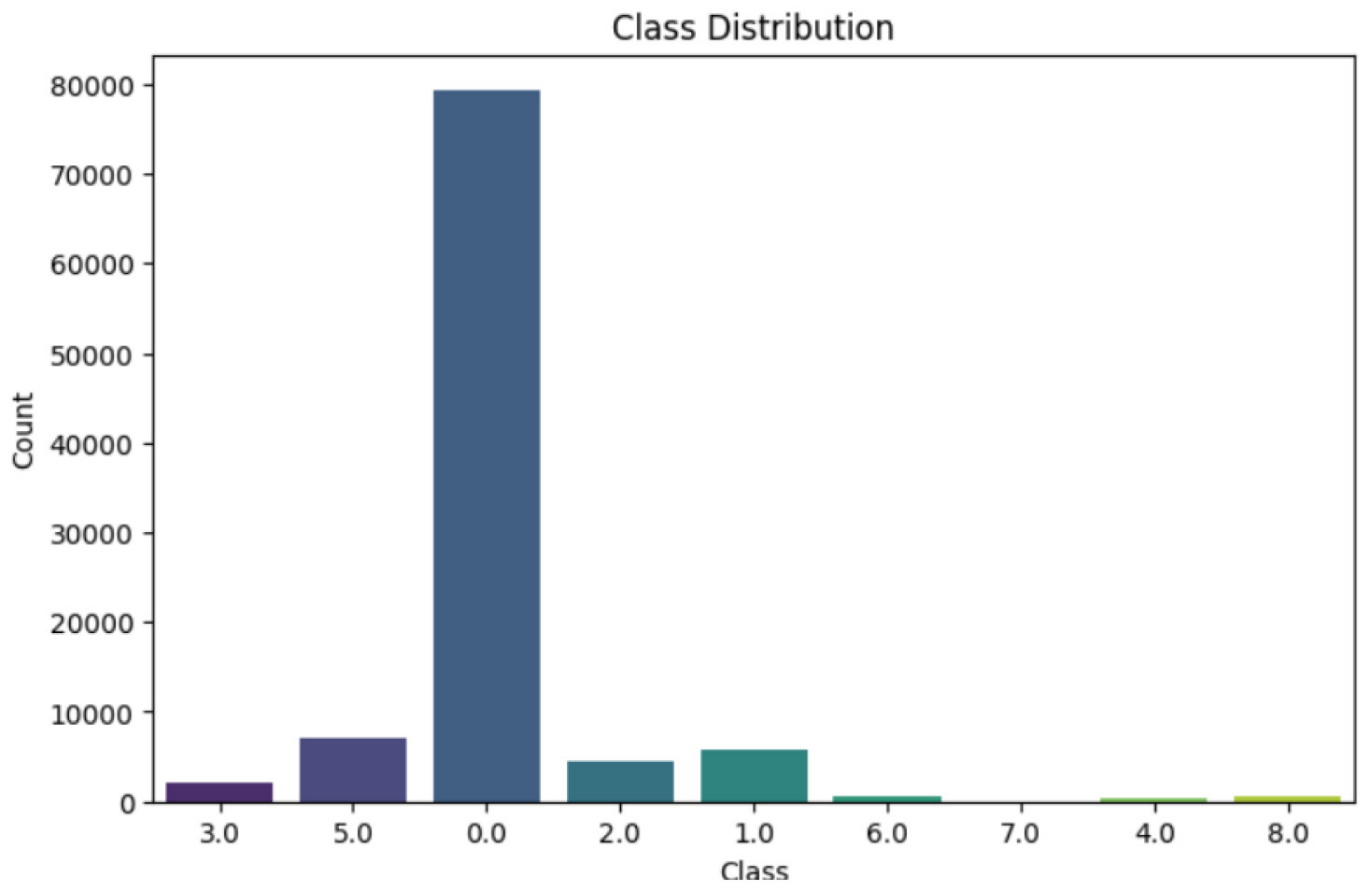
Overall class distribution of the Kitsune Network attack data.

**Figure 6:**
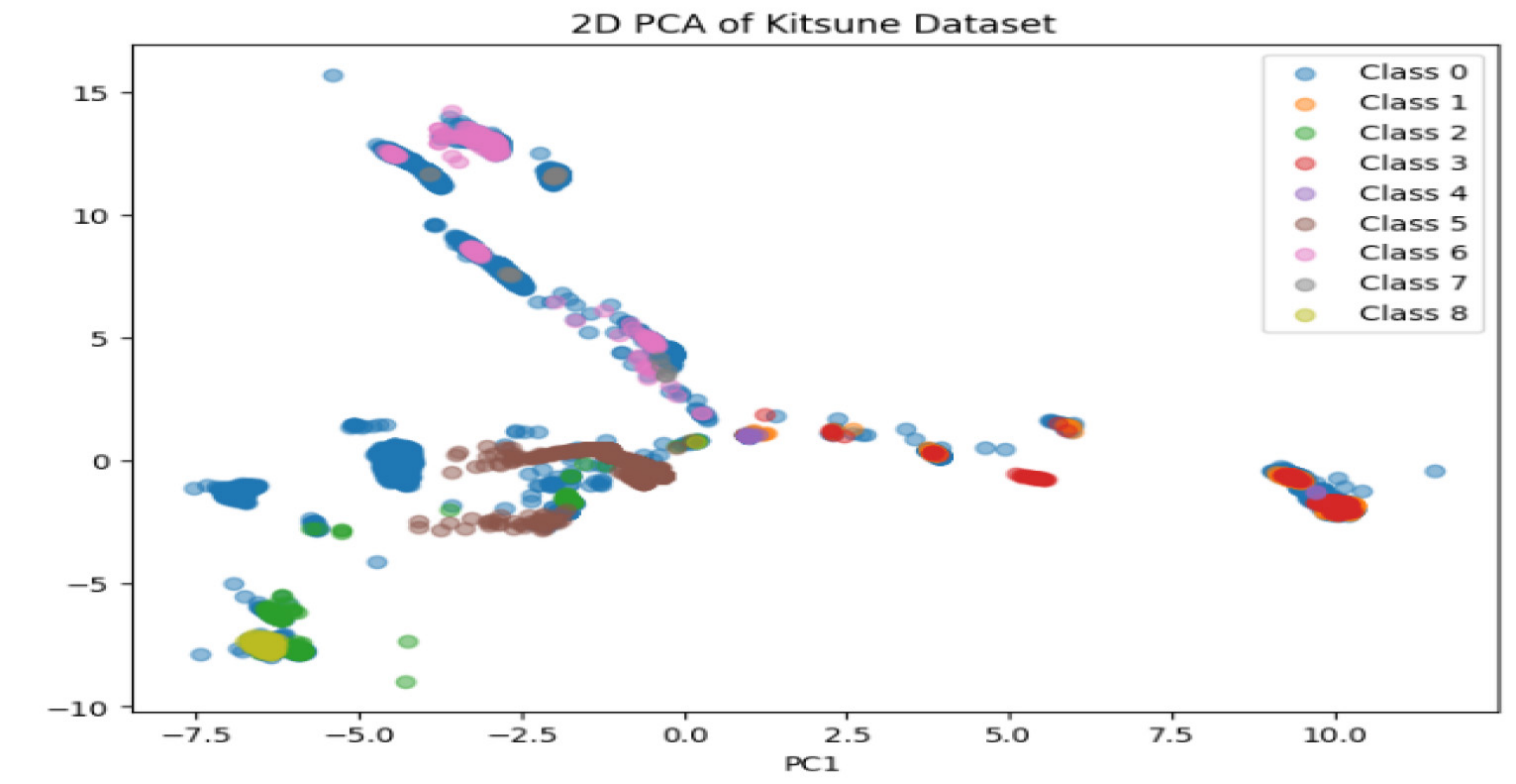
Clusters of Every Class of Kitsune Network attack dataset.

The Kitsune Network Attack dataset (Kitsune Network Attack, 2019) contains over 27 million samples, each represented by 115 statistical features mined from network traffic. These features included metrics like packet rate, inter-arrival time, channel-level statistics, and socket-level statistics across multiple temporal windows. The dataset includes both normal and malicious traffic across nine distinct attack types, namely Fuzzing, Operating System (OS) Scan, SYN DoS, Address Resolution Protocol (ARP) man-in-the-middle, Video Injection, SSDP Flood, SSL Renegotiation, Active Wiretap, and Mirai botnet, which reflect real-world network intrusion scenarios. Period-based feature extraction aligns with security violations across the CIA triad, making this dataset highly suitable for advanced intrusion-detection research. The overall sample of each class and the clusters of each class of the Kitsune Network attack dataset are shown in Figures 5, 6.

### 4.3 Hyperparameter tuning

The attention-enhanced 1D CNN, LSTM, and GRU models were fine-tuned by adjusting various hyperparameters like dropout rate, number of epochs, batch size, learning rate, and network units. The model employed the Adam optimizer to provide stable convergence and effective gradient updates. Dropout layers ranging from 20 to 30% were incorporated at different stages of the models to reduce overfitting and improve generalization. The attention-enhanced 1D CNN, LSTM, and GRU models were trained for 50 epochs using a constant learning rate of 0.001 and a batch size of 128.

### 4.4 Evaluation metrics

The performance of the proposed attention-enhanced 1D CNN, LSTM, and GRU models for IoT network intrusion detection was evaluated using metrics, such as accuracy, precision, recall, F1-score, and a confusion matrix. Testing accuracy was determined by comparing the predicted labels on the test dataset with the actual class labels. The metrics are calculated as given in Equations 18–21.


$$\begin{array}{lll} \text{Accuracy} & = & \frac{\text{TP}+\text{TN}}{\text{TP}+\text{TN}+\text{FP}+\text{FN}} \end{array}$$ (18)

$$\begin{array}{lll} \text{Precision} & = & \frac{\text{TP}}{\text{TP}+\text{FP}} \end{array}$$ (19)

$$\begin{array}{lll} \text{Recall} & = & \frac{\text{TP}}{\text{TP}+\text{FN}} \end{array}$$ (20)

$$\begin{array}{lll} \text{F}1-\text{Score} & = & \frac{2\;*\text{ Precision }*\text{ Recall}}{\text{Precision}+\text{Recall}} \end{array}$$ (21)

Where *TP* represents true positives, *FP* false positives, *TN* true negatives and *FN* false negatives. True positives and true negatives show correctly identified instances of each class, while false positives and false negatives represent misclassifications. The confusion matrix visualizes class-wise performance to identify potential misclassifications. These metrics collectively provide a comprehensive evaluation to detect different types of IoT network attacks. The ability to distinguish between multiple attack classes was evaluated using multi-class ROC curves and the area under the ROC curve for each class. The ROC analysis provides insight into the sensitivity and specificity of the models across all classes, showing a comprehensive assessment of their detection capability in IoT network traffic.

### 4.5 Model training and validation

The classification performance of the attention-enhanced 1D CNN, LSTM, and GRU models was evaluated by training and testing them on Kitsune and HCRL IoT network datasets. The attention-enhanced 1D CNN architecture comprises multiple convolutional layers integrated with channel and spatial-attention blocks, followed by dense layers that capture both local and global features. The LSTM and GRU models used recurrent layers to learn temporal dependencies from sequential network traffic, followed by dense layers for classification.

All three models were trained using the Adam optimizer over 50 epochs with a batch size of 128. Dropout layers ranging from 20 to 30% were applied to reduce overfitting and improve generalization. Training and validation accuracy and loss curves for each model are illustrated in Tables 3, 4. The training loss decreased steadily while training accuracy increased correspondingly. After a few iterations, the improvement rate gradually slowed, indicating convergence and stabilization of the learning process.

**Table 3:** Accuracy and loss plot for HCRL dataset.

Model	Loss	Accuracy
Attention-enhanced 1D CNN	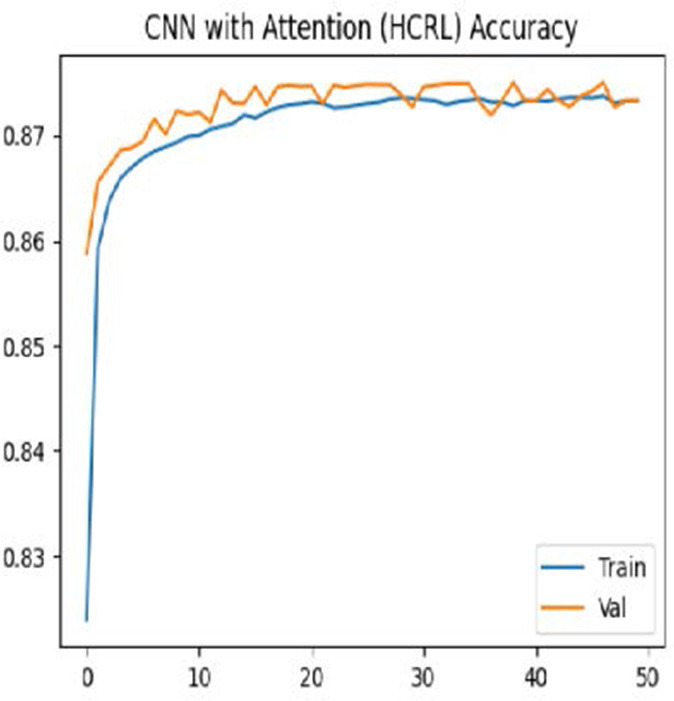	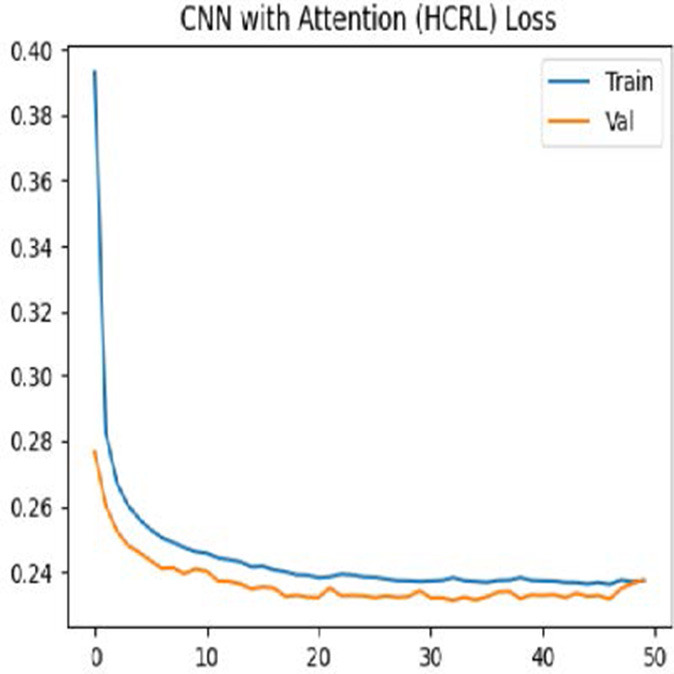
LSTM	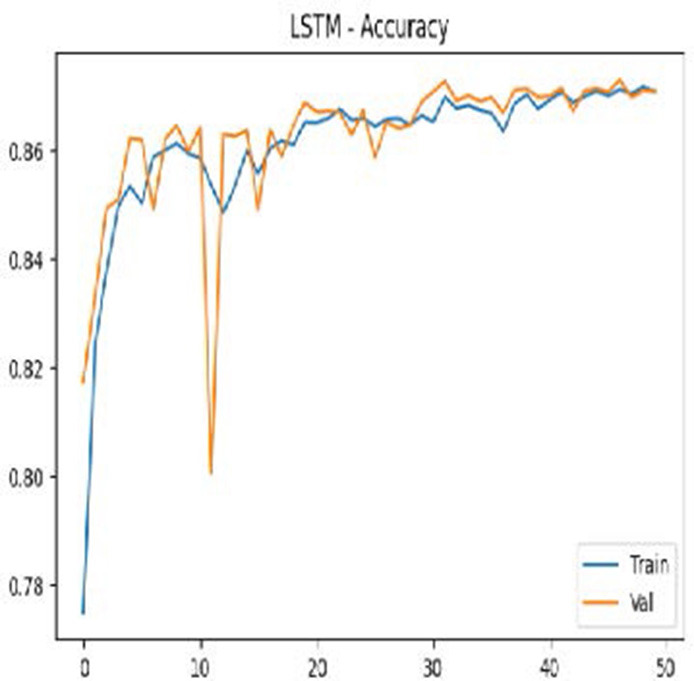	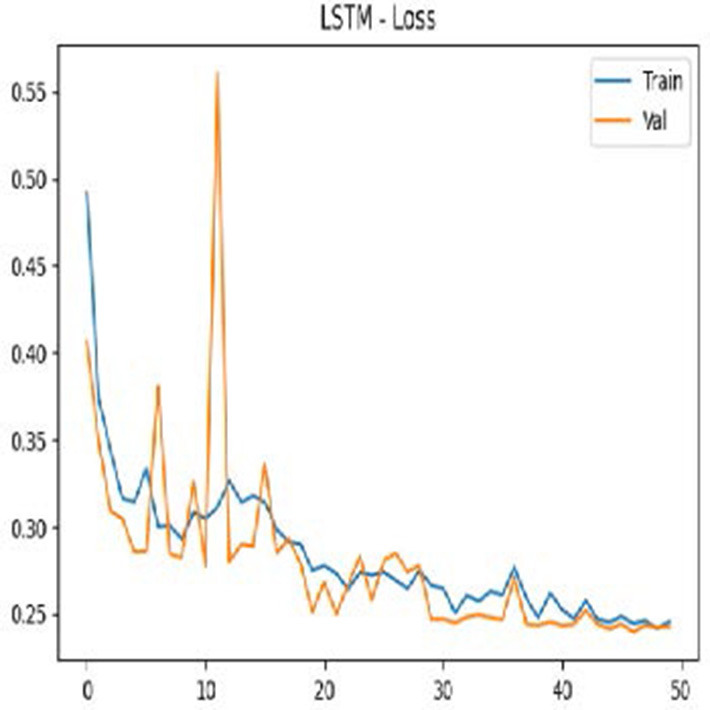
GRU	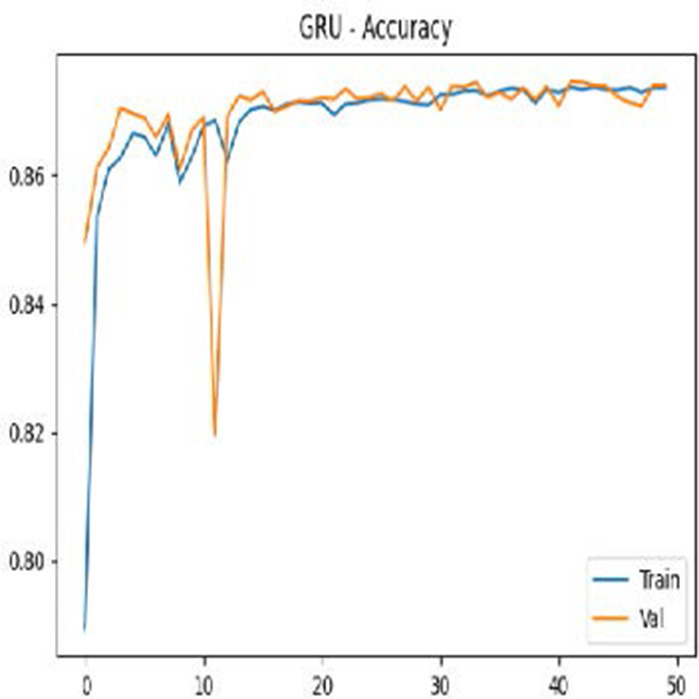	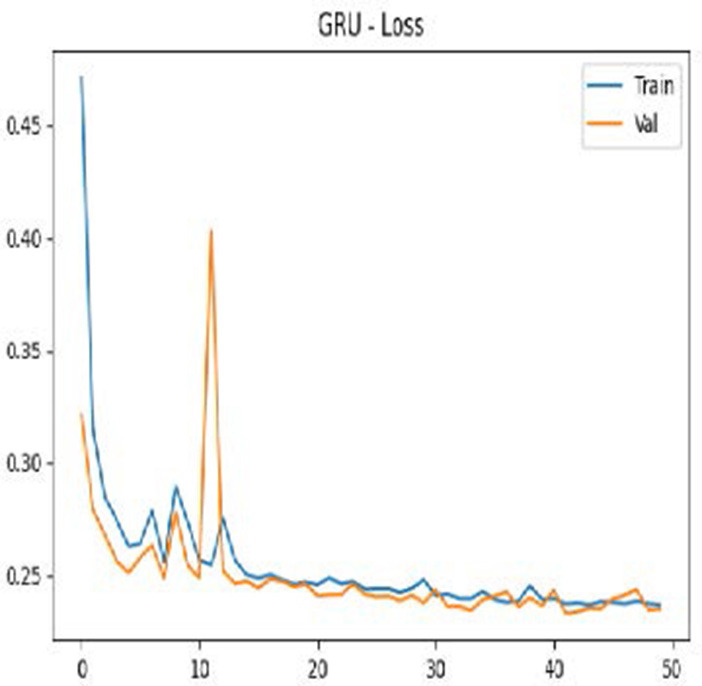

**Table 4:** Accuracy and loss plot for Kitsune dataset.

Model	Loss	Accuracy
Attention-enhanced 1D CNN	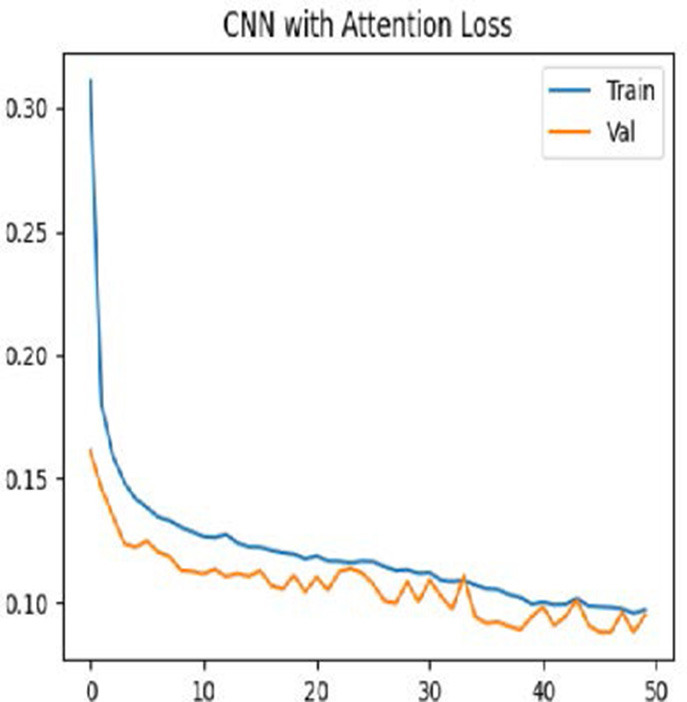	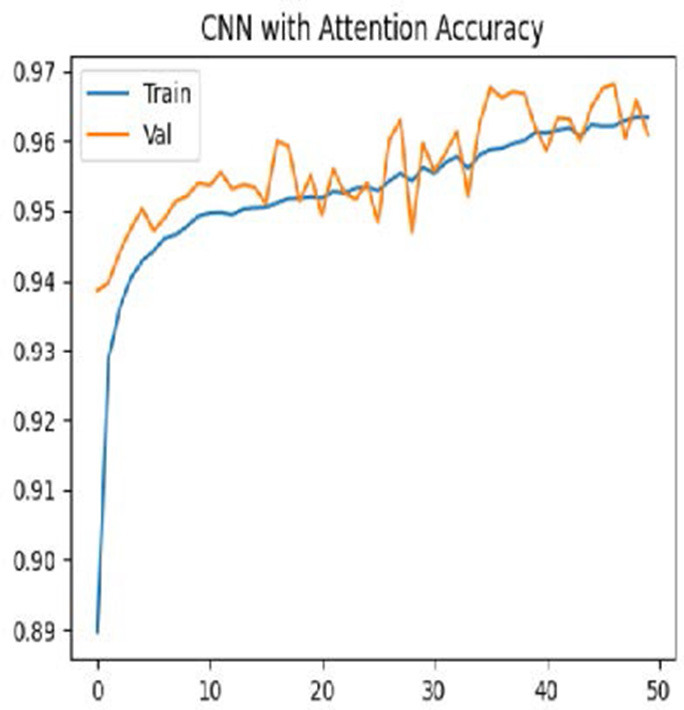
LSTM	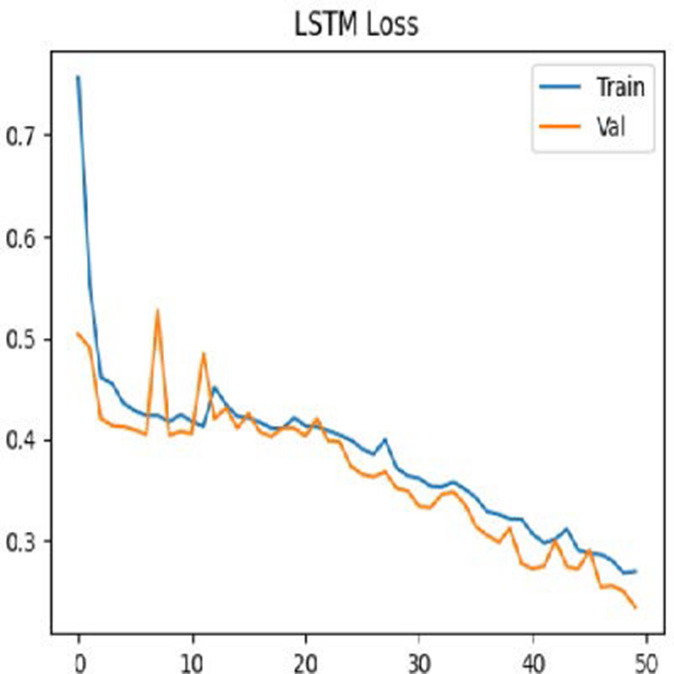	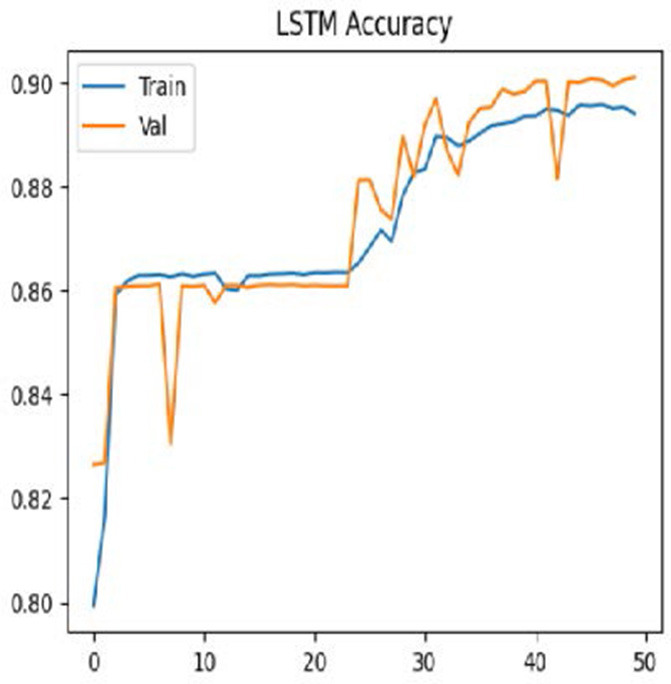
GRU	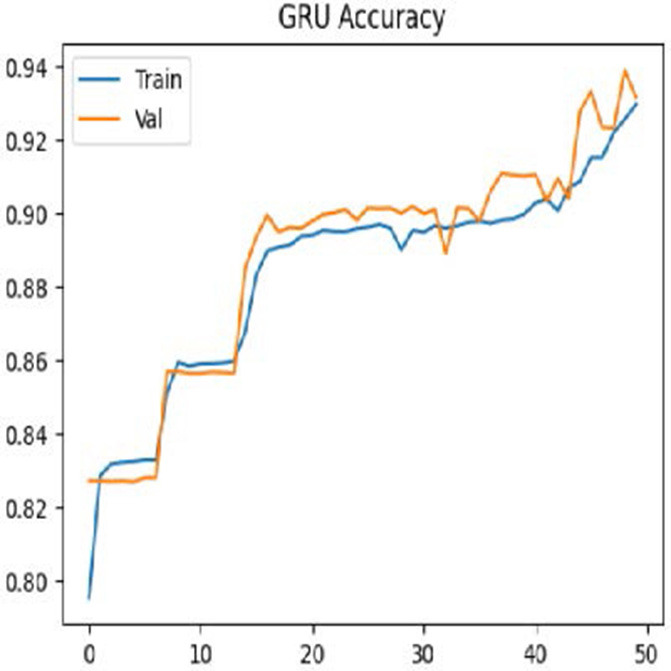	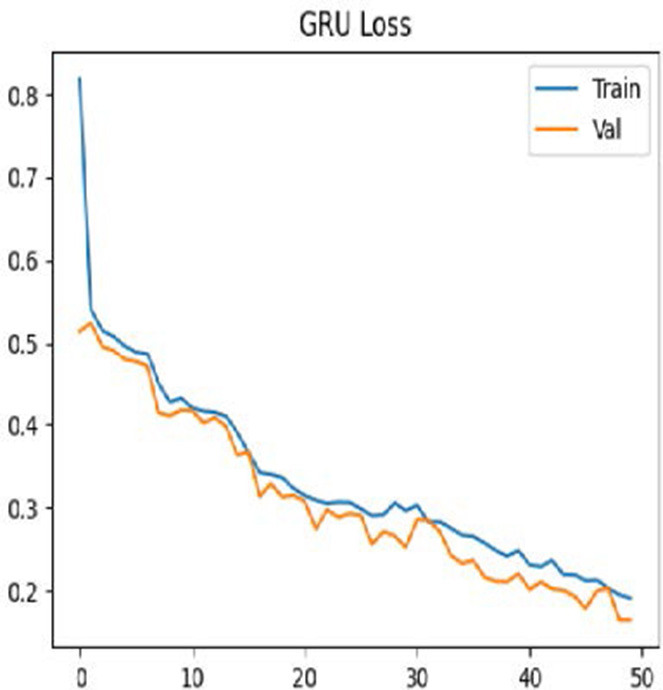

The attention-enhanced 1D CNN demonstrated faster convergence and higher overall accuracy than the recurrent models, demonstrating its ability to extract discriminative features from network traffic. LSTM and GRU models captured temporal correlations and performed better against attacks with sequential patterns, although with slightly lower accuracy against static attack types. Batch normalization and dropout contributed to stable learning and mitigated overfitting, while the learning rate of 0.001 ensured consistent and efficient convergence. Attention-enhanced 1D CNN, LSTM, and GRU models are computationally efficient with fewer trainable parameters compared to more complex architectures, allowing faster training and effective detection of diverse IoT network attacks across multiple classes. The dataset is divided using an 80:20 ratio for train and test split, with 10% of the training data used as a validation. No explicit resampling techniques were applied; instead, stratified splitting was used to preserve the dataset's natural class distribution. Internet Protocol (IP) addresses were decomposed into four numerical octets each, protocol types were encoded using one-hot encoding, and numerical features were normalized using standard scaling. The attention-enhanced 1D Convolutional Neural Network (CNN), Long Short-Term Memory (LSTM), and Gated Recurrent Unit (GRU). All models use the Adam optimizer, a learning rate of 0.001, batch size of 512, and are trained for 50 epochs. A fixed random state of 42 was used for dataset splitting to ensure reproducibility.

### 4.6 Performance analysis of the proposed system

#### 4.6.1 HCRL dataset

The performance of the proposed attention-enhanced 1D CNN, LSTM, and GRU models was evaluated on the HCRL dataset using various classification metrics. Table 5 summarizes the results for the three major classes: Benign, DoS, and Mirai. The attention-enhanced 1D CNN model achieved an overall accuracy of 87%. It showed high precision and recall for the DoS class with 1.00 and 0.95, respectively. The Mirai class achieved precision of 0.86 and a recall of 1.00, indicating strong detection of malicious traffic. The Benign class exhibited a lower recall of 0.08, despite a precision of 0.84, showing occasional misclassification of normal traffic as attacks. The LSTM model achieved an overall accuracy of 87.14%, with better DoS detection precision of 0.99 and recall of 0.95. Mirai attack class attained a precision of 0.85 and a recall of 0.99, where the Benign class had a lower recall of 0.07 while maintaining a moderate precision of 0.78. The GRU model achieved an overall accuracy of 87% with high precision and recall for DoS of 0.99 and 0.96, respectively. Mirai was classified with a precision of 0.85 and a recall of 0.99, with the Benign class having a lower recall of 0.08 while maintaining precision at 0.78. All three models successfully detected malicious traffic, particularly DoS and Mirai attacks. The Benign class remained the most challenging to classify accurately. Among the models, the attention-enhanced 1D CNN slightly outperformed LSTM and GRU in overall accuracy and F1-score, demonstrating the advantage of attention-based convolutional feature extraction for IoT intrusion detection. Despite the high overall accuracy, further improvements are needed to increase benign traffic detection. The confusion matrix and the ROC plot for the HCRL dataset are shown in Table 6.

**Table 5:** Performance comparison of the proposed models for the HCRL dataset.

HCRL dataset	Class	Precision	Recall	F1-score	Accuracy
1DCNN	Benign	0.84	0.08	0.15	0.8739
	DoS	1.00	0.95	0.98	
	Mirai	0.86	1.00	0.92	
LSTM	Benign	0.78	0.07	0.14	0.8714
	DoS	0.99	0.95	0.97	
	Mirai	0.85	0.99	0.92	
GRU	Benign	0.78	0.08	0.15	0.8743
	DoS	0.99	0.96	0.98	
	Mirai	0.85	0.99	0.92	

**Table 6:** Confusion matrix and the ROC plot for the HCRL dataset.

HCRL dataset		
Model	Confusion matrix	ROC plot
Attention-enhanced 1D CNN	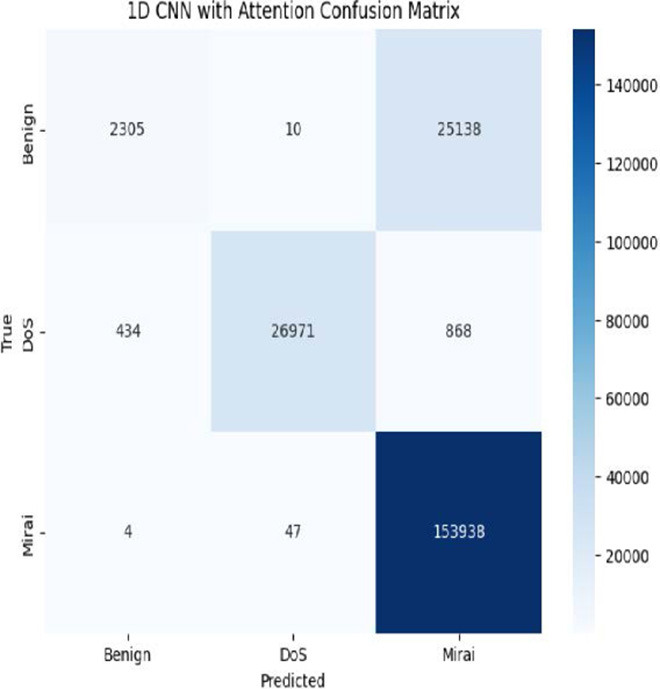	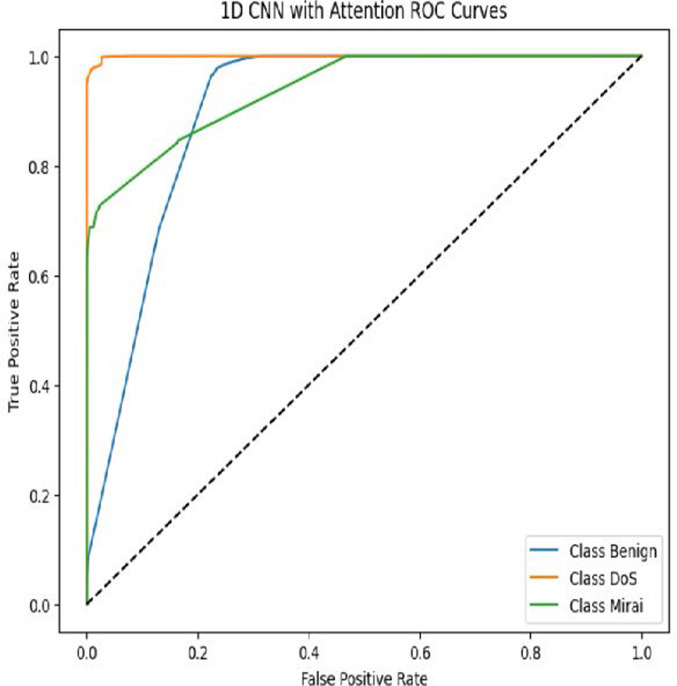
LSTM	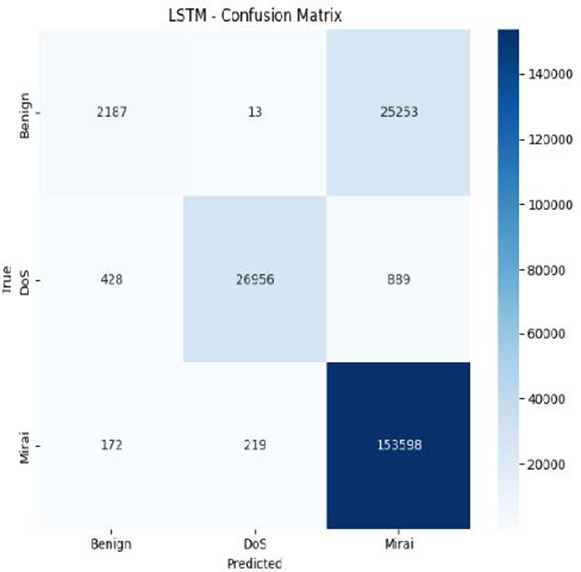	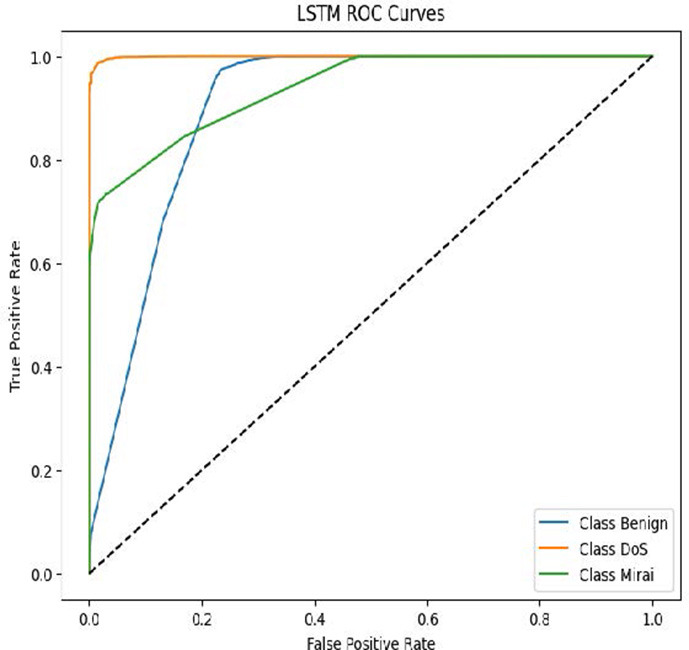
GRU	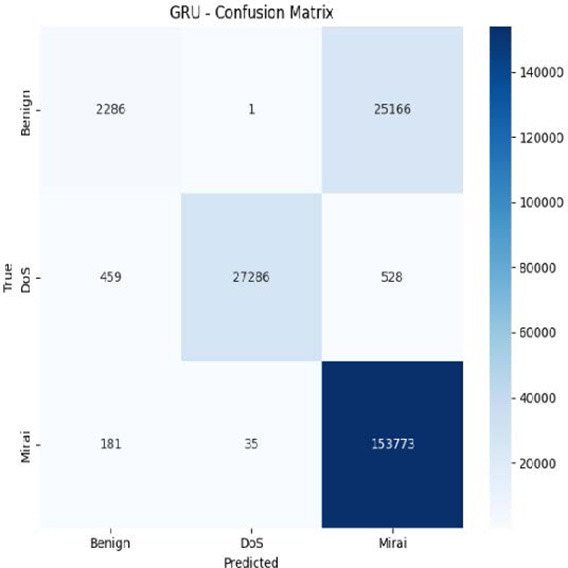	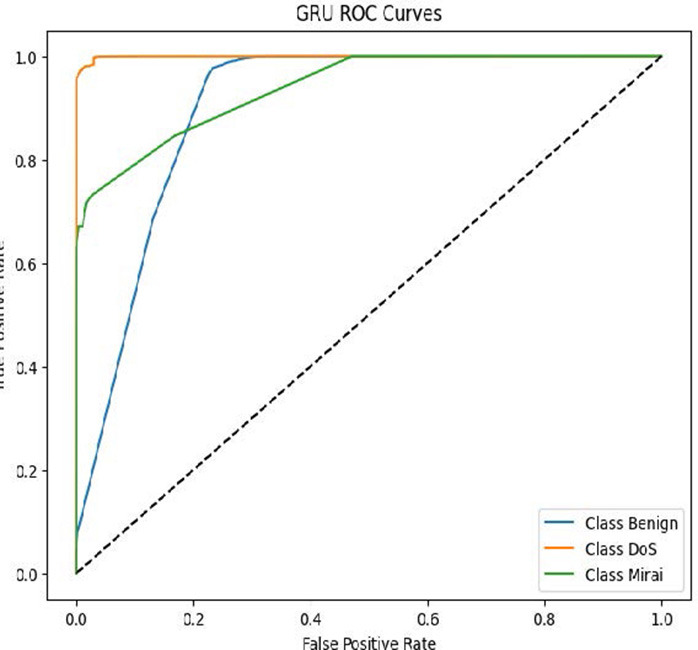

Class imbalance in HCRL data is addressed by balanced class weighting during model training, which penalizes misclassifications of the minority benign class, significantly improving its recall from approximately 0.07 to 0.96 in the improved version. To improve training stability and prevent overfitting, Early Stopping and ReduceLROnPlateau callbacks are incorporated, ensuring optimal convergence. Balanced Accuracy and Matthews correlation coefficient (MCC) are included for imbalanced conditions. Per-class recall values are presented to show the class-wise performance for the benign class, providing transparency into how each model handles each traffic category in Tables 7–9.

**Table 7:** Accuracy and loss plot with class weighting and early stopping for HCRL dataset.

Model	Loss	Accuracy
Attention-enhanced 1D CNN	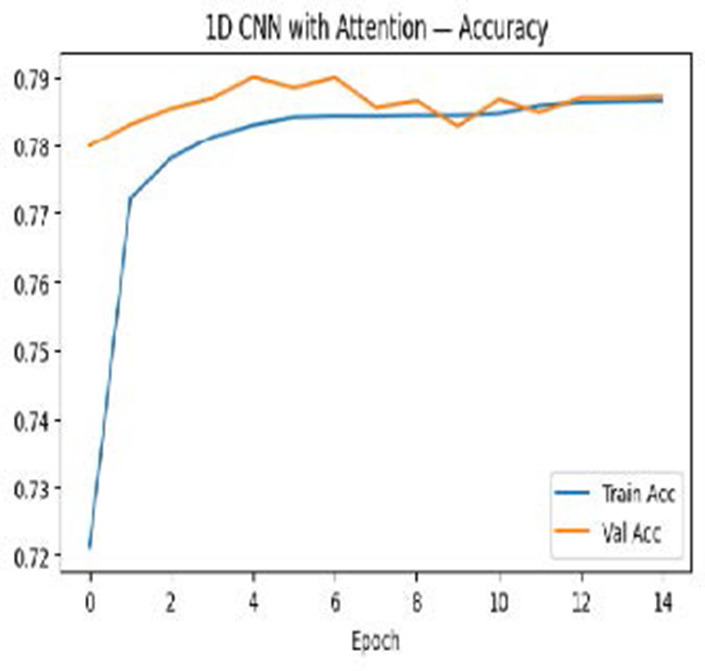	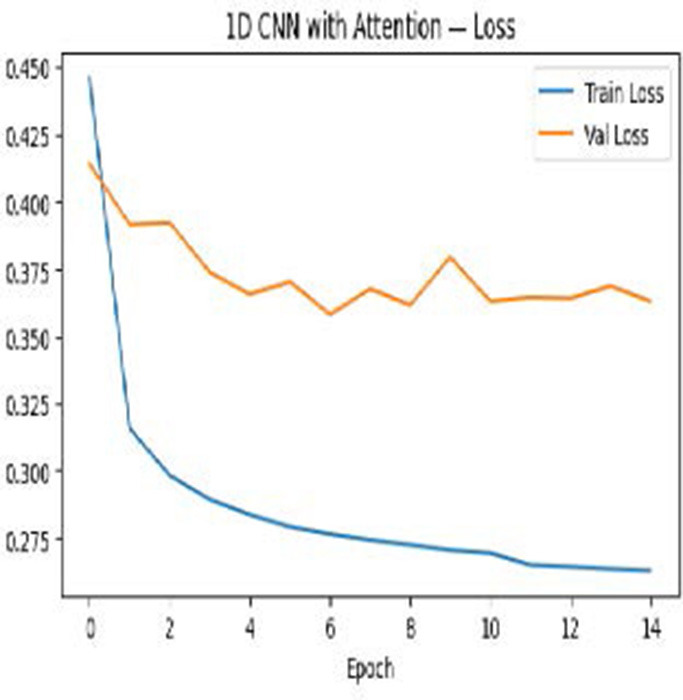
LSTM	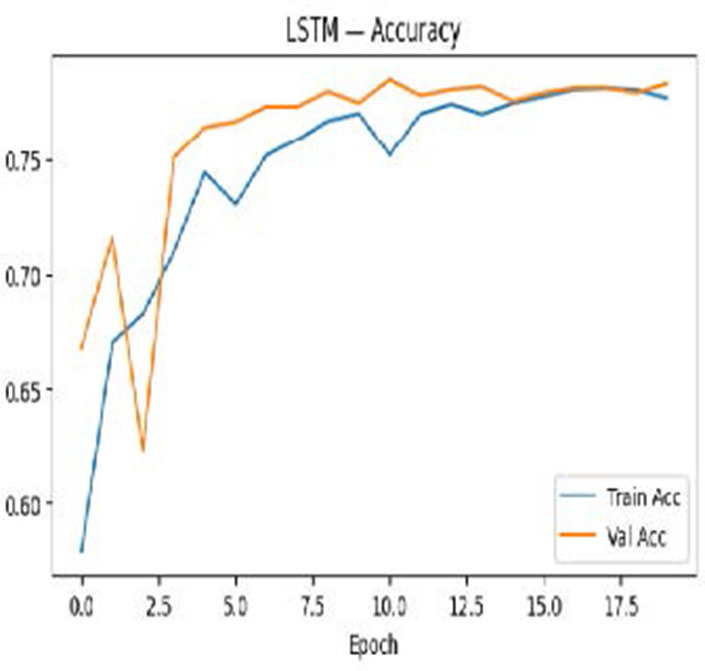	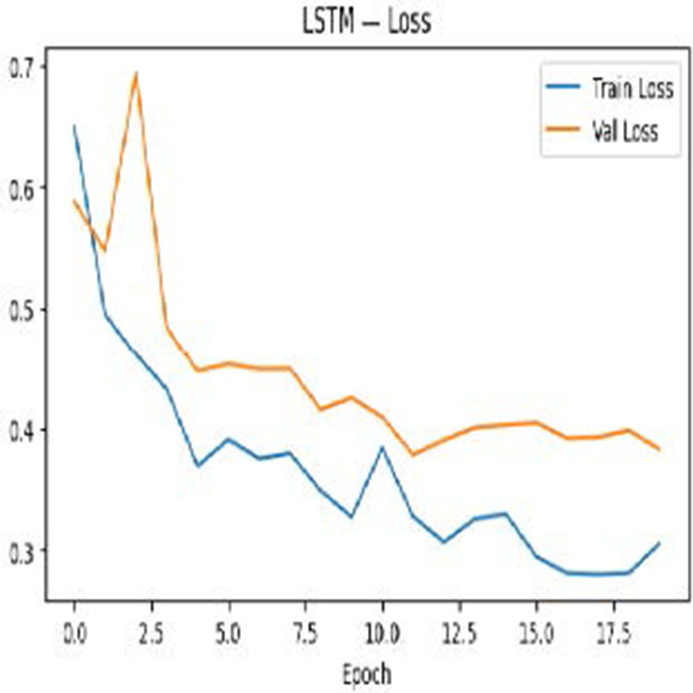
GRU	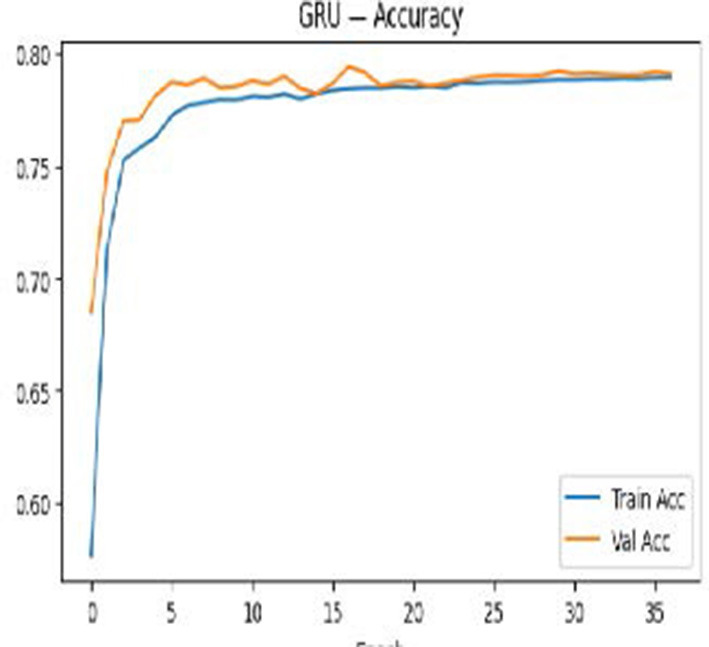	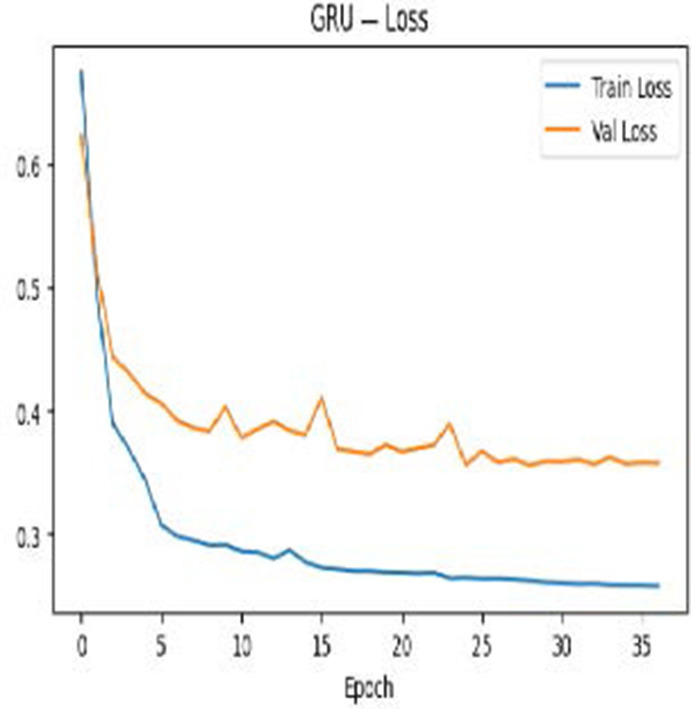

**Table 8:** Performance comparison of the proposed models with class weighting and early stopping for the HCRL dataset.

HCRL dataset	Class	Per-class recall (%)	Balanced accuracy (%)	Matthews correlation coefficient (MCC) (%)
Attention-enhanced 1D CNN	Benign	0.96	0.88	0.66
	DoS	0.97		
	Mirai	0.72		
LSTM	Benign	0.96	0.87	0.65
	DoS	0.95		
	Mirai	0.70		
GRU	Benign	0.97	0.89	0.67
	DoS	0.97		
	Mirai	0.72		

**Table 9:** Confusion matrix and the ROC plot models with class weighting and early stopping for the HCRL dataset.

HCRL Dataset		
Model	Confusion matrix	ROC plot
Attention-enhanced 1D CNN	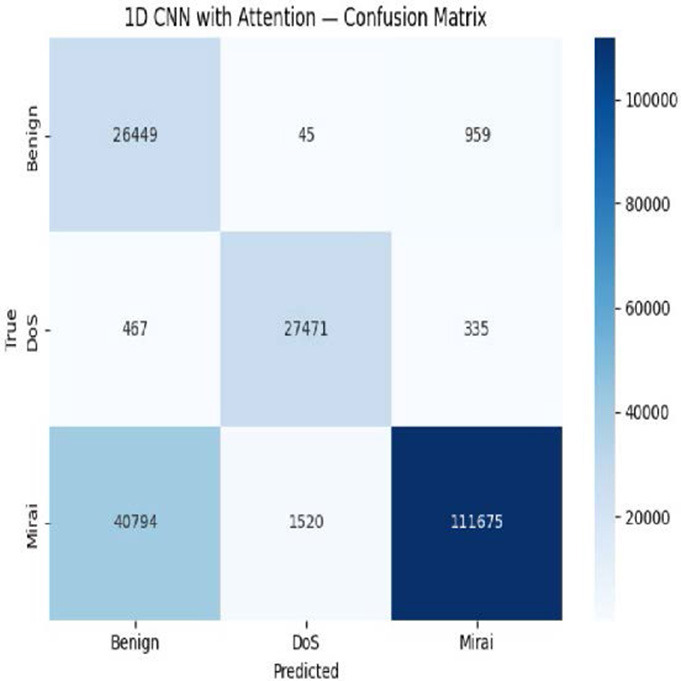	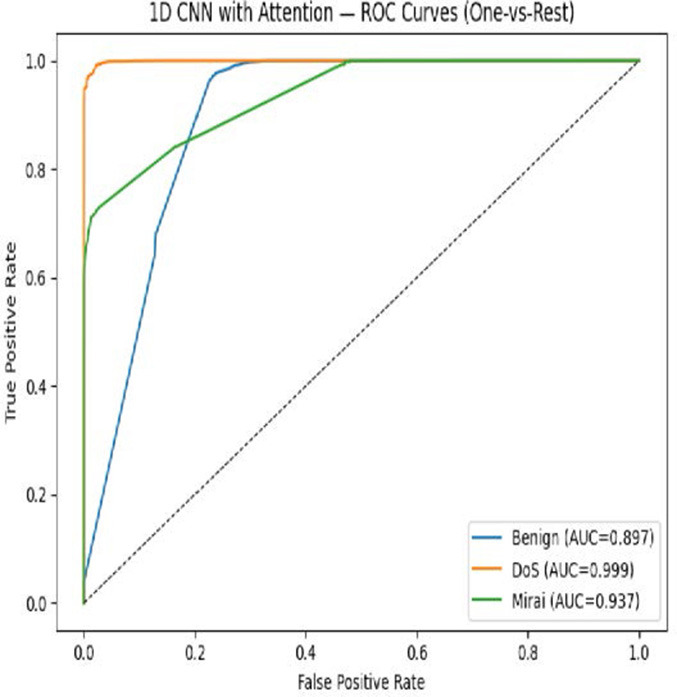
LSTM	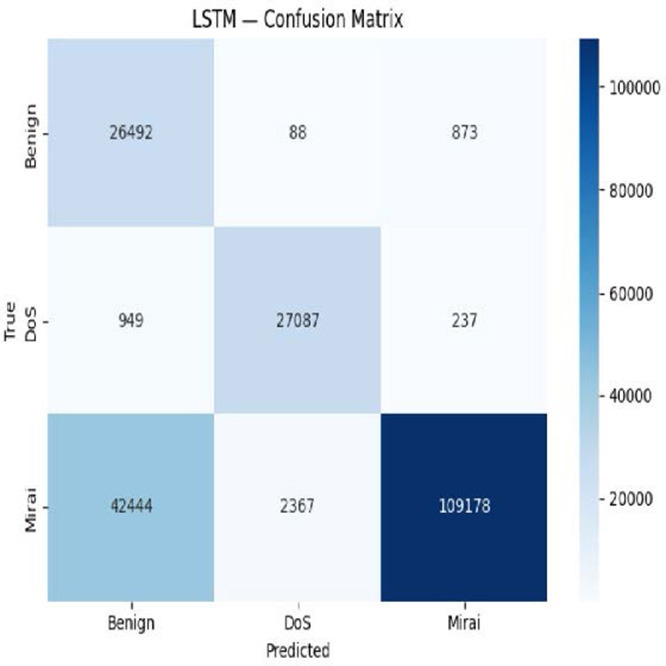	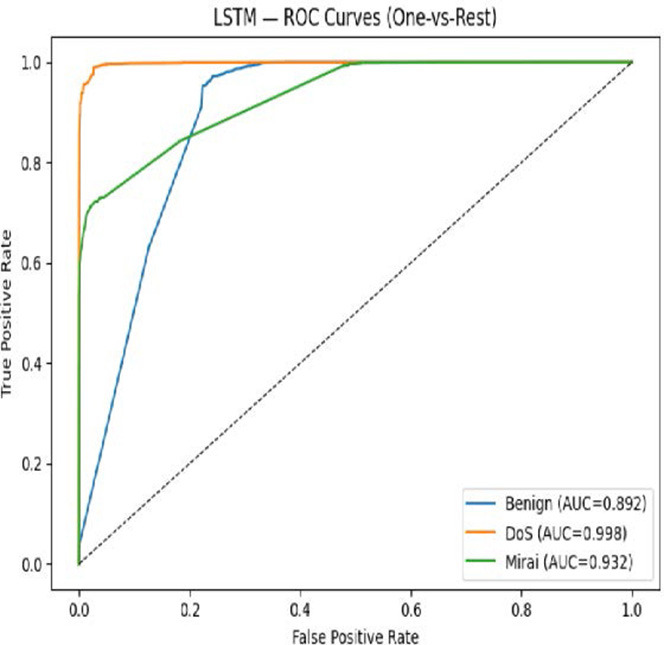
GRU	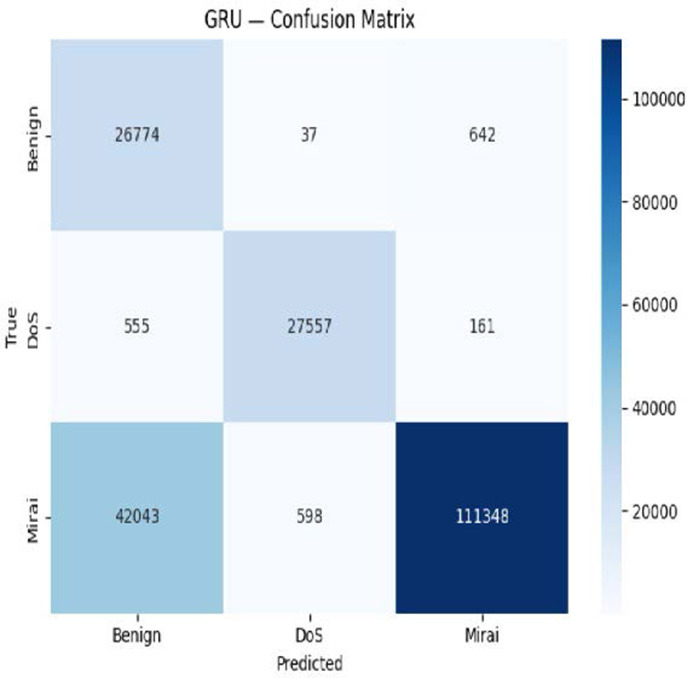	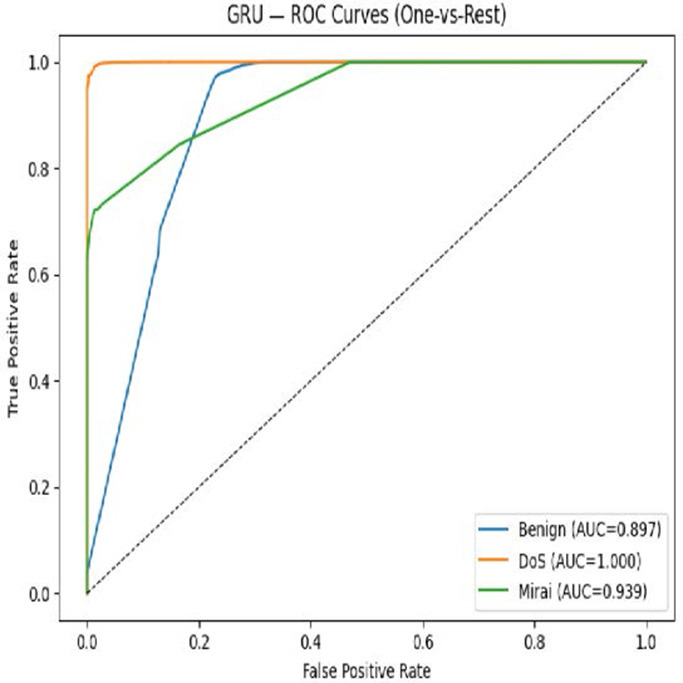

#### 4.6.2 Kitsune dataset

The attention-enhanced 1D CNN, LSTM, and GRU models were evaluated on the Kitsune dataset using various metrics, as shown in Table 10. Attention-enhanced 1D CNN achieved the highest overall accuracy of 96%, demonstrating strong detection across several attack categories. F1-scores obtained for Classes 0–3, 5, and 8 are 0.98, 0.91, 0.95, 0.75, 0.99, and 0.77, respectively. However, the model struggled with Classes 4–7, showing lower recall value. The LSTM model achieved an overall accuracy of 90% and performed well on Classes 0 and 5, obtaining an F1-score of 0.94 and 0.99, but completely failed to detect multiple classes, specifically Classes 2, 3, 4, 6, 7, and 8. This indicates that LSTM had difficulty modeling the separability of minority or overlapping classes in the Kitsune dataset. The GRU model achieved an overall accuracy of 93% with strong detection for Classes 0–3 and 5 with F1-score as 0.96, 0.80, 0.74, 0.72, and 0.99. Overall, attention-enhanced 1D CNN demonstrated the better performance with the highest accuracy and balanced detection across the majority of the attack types. LSTM and GRU performed well for major attacks, but both experienced difficulty with minority or highly similar traffic classes. The confusion matrix and the ROC plot obtained for the Kitsune Network dataset are shown in Table 11.

**Table 10:** Performance Comparison of the proposed models for the Kitsune dataset.

Kitsune dataset	Class	Precision	Recall	F1-score	Accuracy
1DCNN	0	0.98	0.97	0.98	0.96
	1	0.86	0.97	0.91	
	2	0.94	0.97	0.95	
	3	0.65	0.88	0.75	
	4	0.00	0.00	0.00	
	5	0.99	1.00	0.99	
	6	0.54	0.41	0.47	
	7	1.00	0.20	0.33	
	8	0.8	0.74	0.77	
LSTM	0	0.89	1	0.94	0.90
	1	0.96	0.68	0.79	
	2	0.00	0.00	0.00	
	3	0.00	0.00	0.00	
	4	0.00	0.00	0.00	
	5	0.99	1	0.99	
	6	0.00	0.00	0.00	
	7	0.00	0.00	0.00	
	8	0.00	0.00	0.00	
GRU	0	0.95	0.96	0.96	0.93
	1	0.95	0.69	0.8	
	2	0.71	0.78	0.74	
	3	0.61	0.88	0.72	
	4	0.00	0.00	0.00	
	5	0.99	1.00	0.99	
	6	0.00	0.00	0.00	
	7	0.00	0.00	0.00	
	8	0.00	0.00	0.00	

**Table 11:** Confusion matrix and the ROC plot for the Kitsune dataset.

Model	Loss	Accuracy
Attention-enhanced 1D CNN	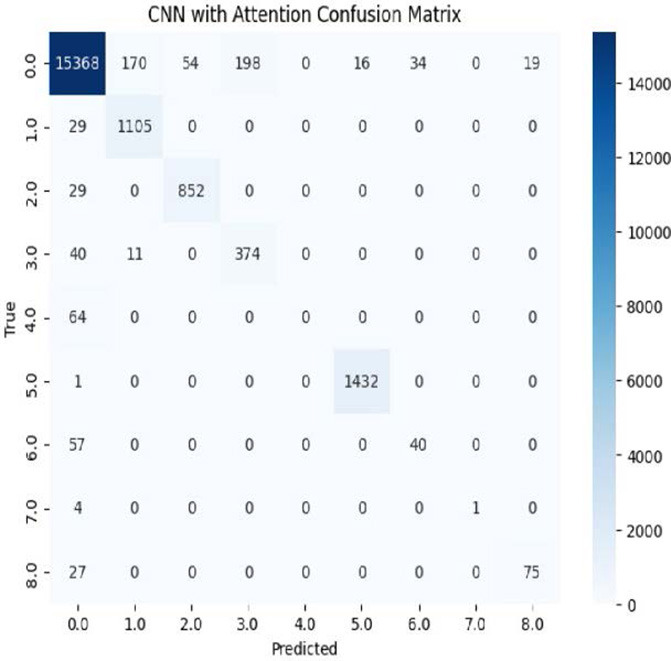	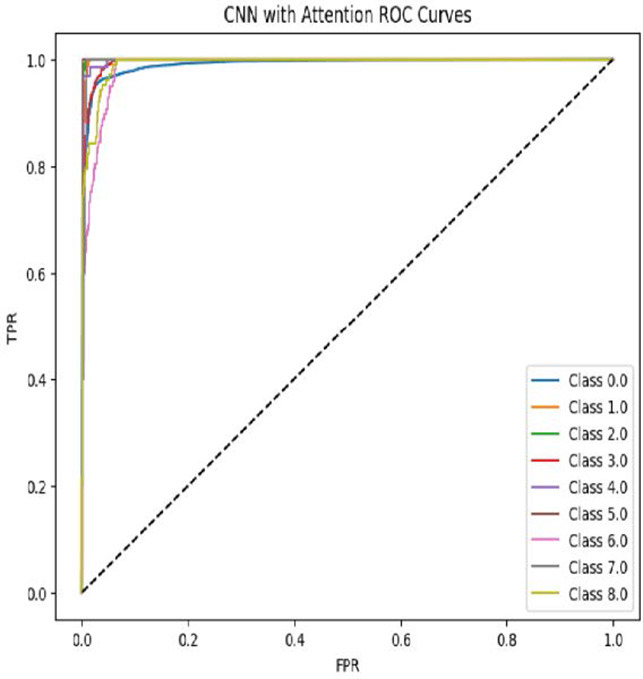
LSTM	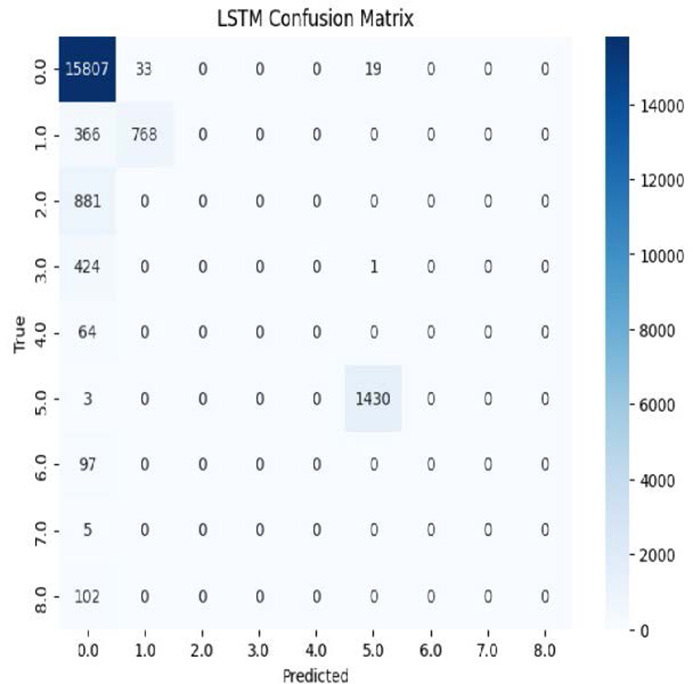	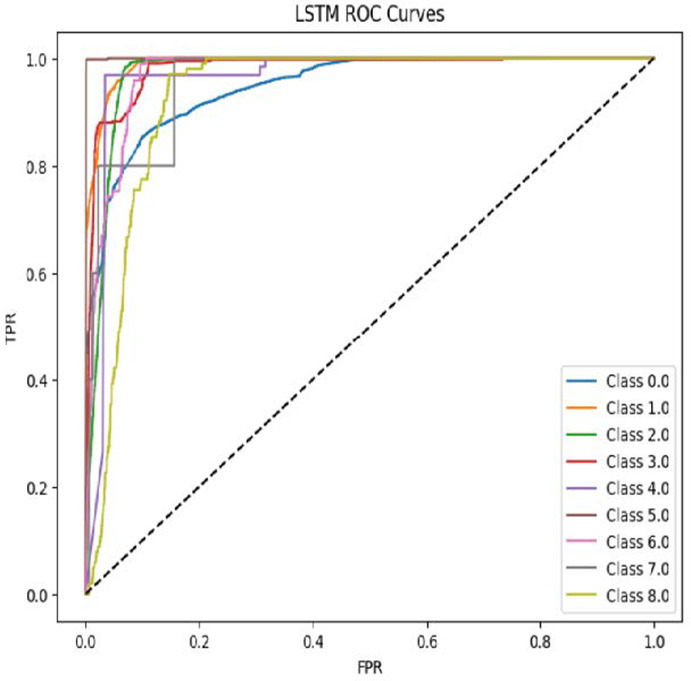
GRU	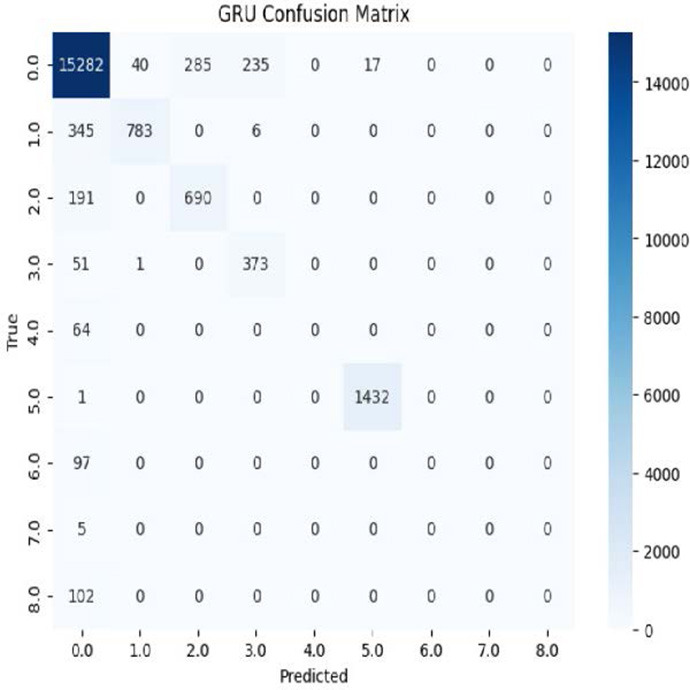	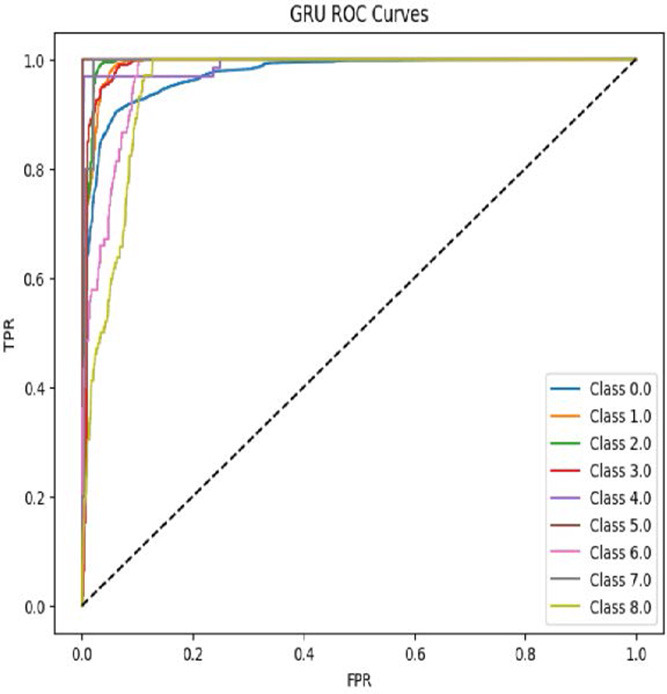

### 4.7 Explainable AI features

The SHapley Additive exPlanations (SHAP) features are a technique used into explain individual predictions to measure the extent to which each feature contributes to the model's output for a particular instance. The SHAP value includes a feature *f* that contributes prediction features as given in Equation 22, where *m* refers to the model analyzed, *y* refers to the feature instances that are explained, *t* refers to the total number of features and *y*_*S*_*u*__ refers to the features instances in the subset *S*_*u*_ related to the background values.


$$\begin{array}{l} {\text{F}}_{\text{f}}(\text{m},\text{y})\text{=}\sum_{\text{u}\subseteq \{\text{1},\text{2},\ldots ..\text{t}\}\backslash \text{f}\}}\frac{{\text{|S}}_{\text{u}}\text{|}!(\text{t }-\;|{\text{S}}_{\text{u}}|\;-\text{ 1})!}{\text{t}!}[\text{m}({\text{y}}_{{\text{S}}_{\text{u}}}\cup \;{\text{y}}_{\text{j}})\\ \;-\text{ m}({\text{y}}_{{\text{S}}_{\text{u}}}) \end{array}$$ (22)

The SHAP values offer valuable insights into the individual contributions of each feature toward the model's output for an IoT intrusion-detection data, thereby facilitating the interpretability and comprehension of the proposed model. SHAP values serve to quantify the incremental impact of individual features on the prediction output. The computation of the SHAP value for a particular feature involves the examination of all conceivable combinations of features, followed by the assessment of the alteration in the model's output when such a feature is incorporated as opposed to its exclusion. Through the analysis of SHAP values, data scientists can determine which features that have the greatest influence on a specific prediction.

The use of SHAP values enhances model debugging, validation, and feature engineering by highlighting the importance of individual features and their impact on overall model performance. SHAP features function as a powerful tool for the interpreting models, offering vital insights into the internal mechanisms of ML models and augmenting trust, transparency, and accountability in the decision making process. The SHAP features obtained for the attention-enhanced 1D CN architecture are shown in Table 12.

**Table 12:** SHAP features obtained for HCRL dataset.

Model	HCRL dataset		
	Benign	Dos	Mirai
Attention-enhanced 1D CNN	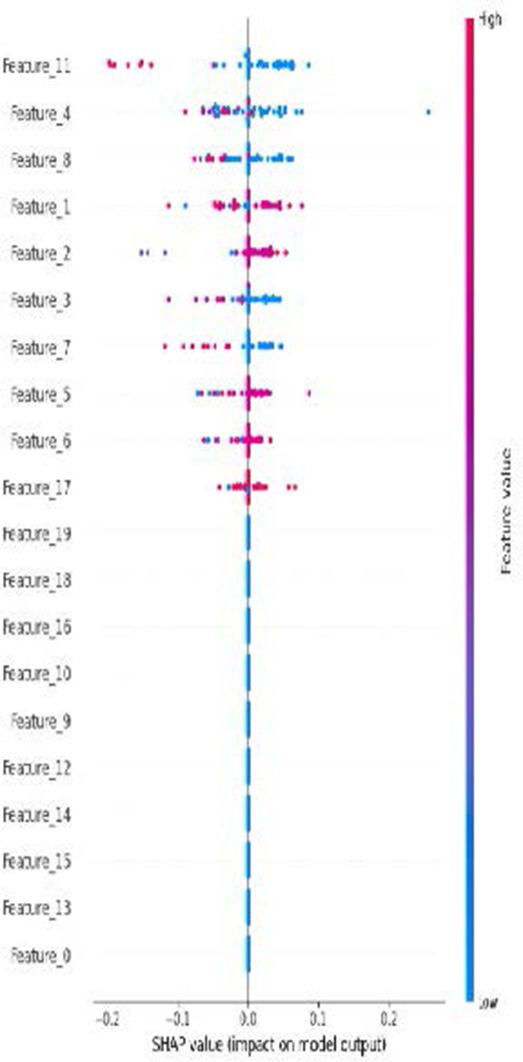	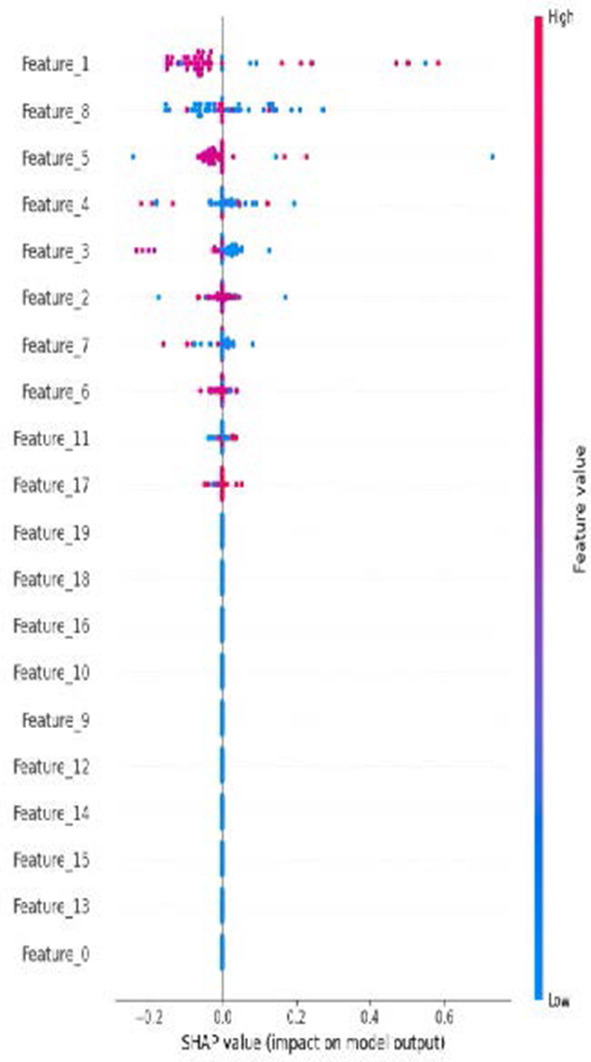	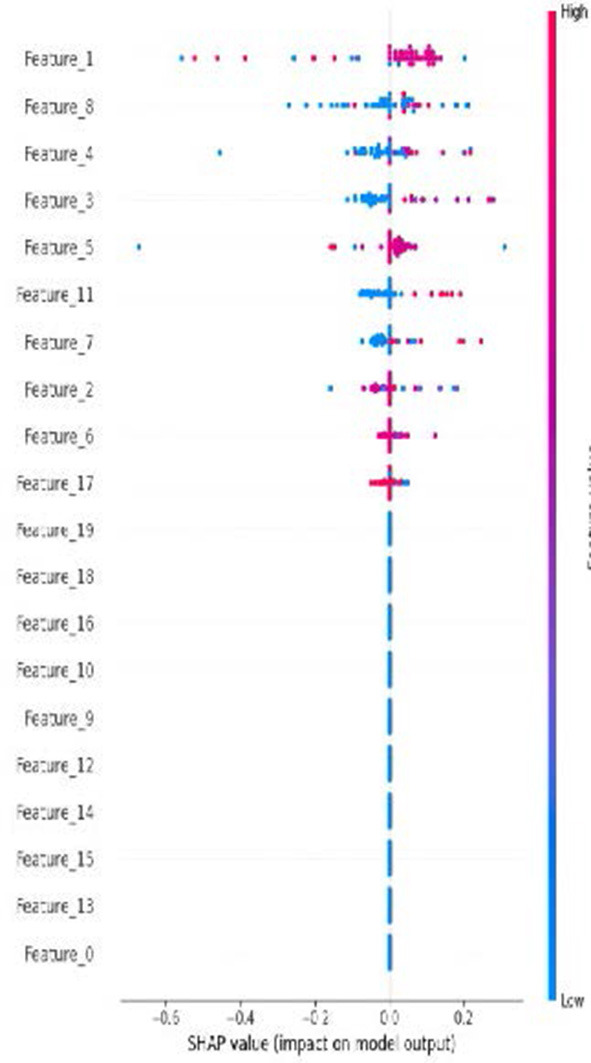
LSTM	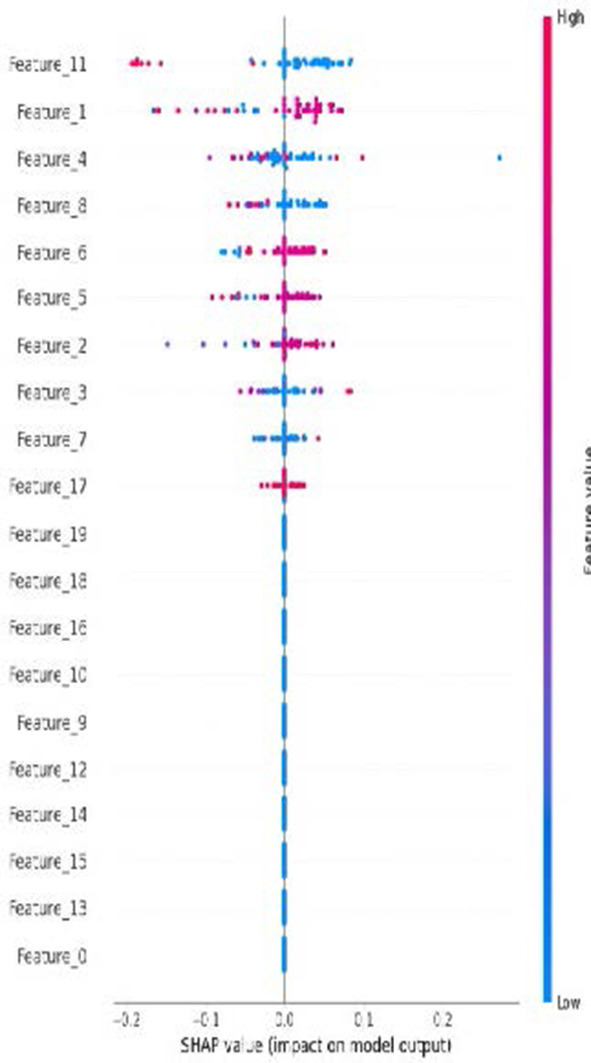	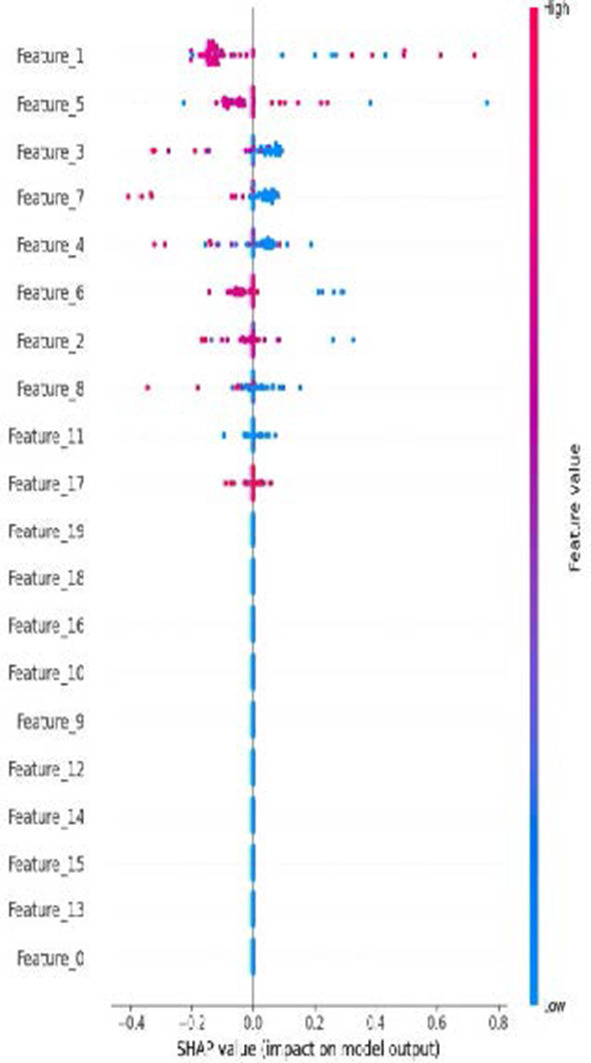	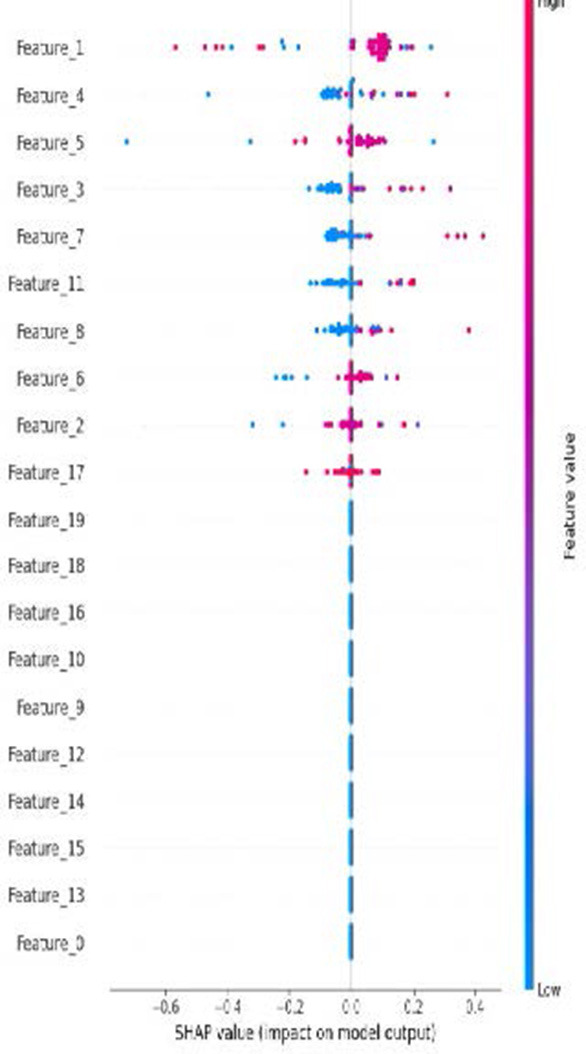
GRU	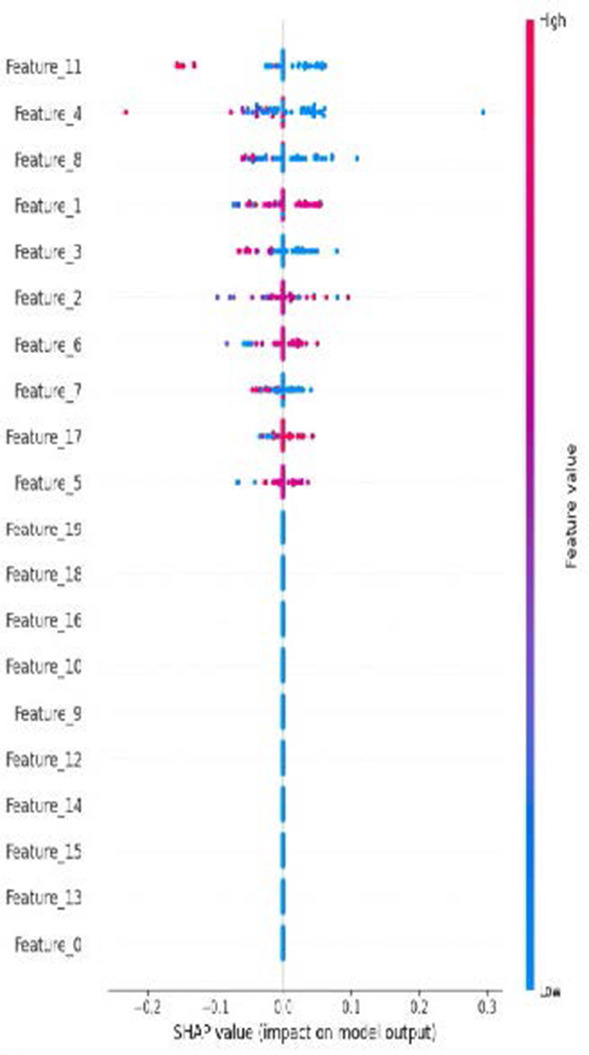	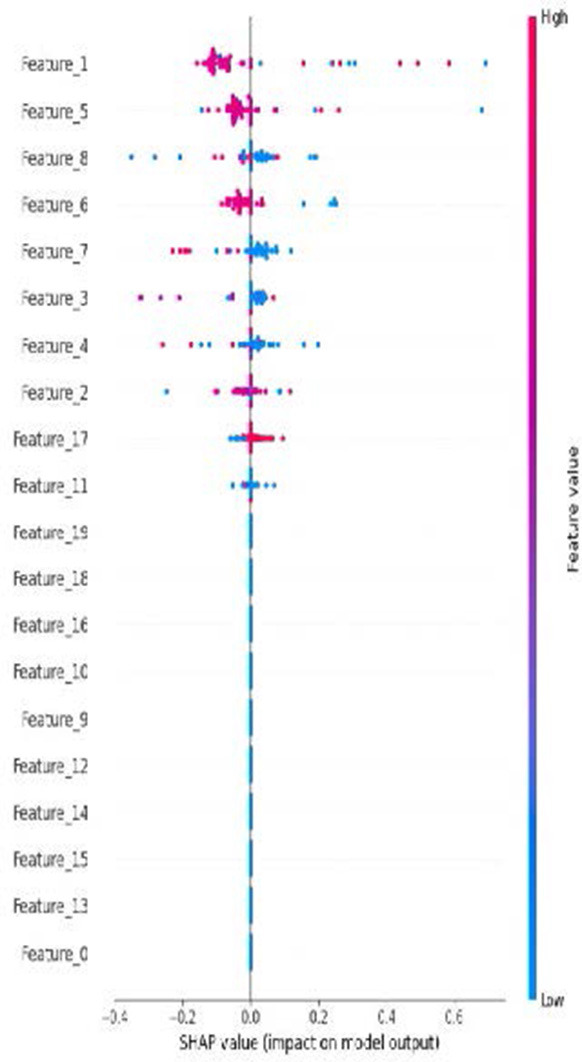	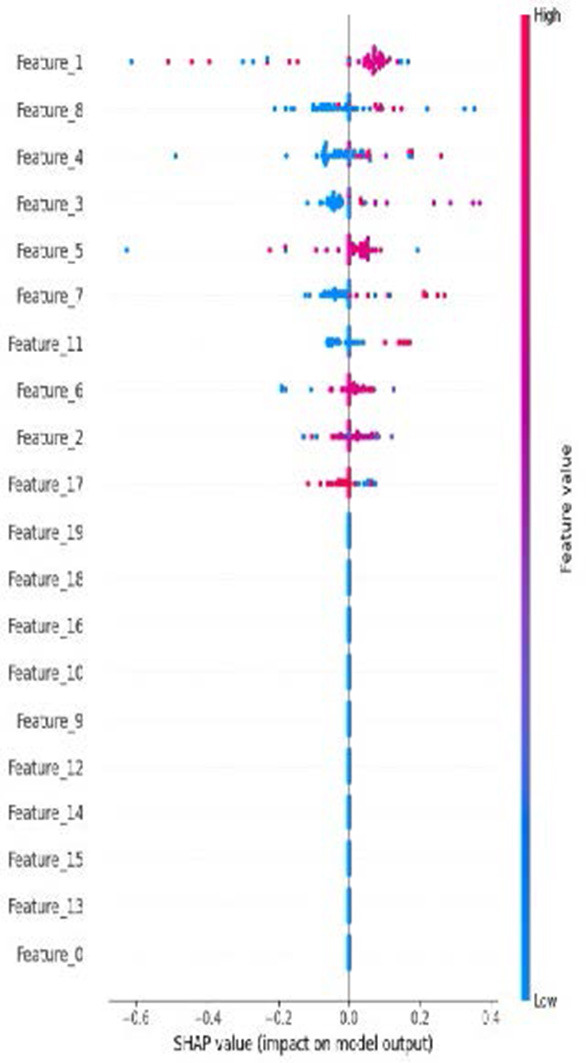

SHAP is applied to interpret attention-enhanced 1D CN, LSTM, and GRU predictions on the HCRL and Kitsune Network Intrusion Dataset, as shown in Table 13. The analysis used 50 representative training samples and considered all network traffic features, including packet length, protocol type, and source/destination IP octets. Inputs were reshaped to match the sequential 3D format required by attention-enhanced 1D CN, LSTM, and GRU models. SHAP summary plots show the contribution of each feature to the prediction of each attack class, with positive values indicating that a feature supports the class prediction and negative values indicating that it suppresses it. This approach provides feature-level interpretability, revealing which traffic attributes most influence model decisions between normal and malicious behavior.

**Table 13:** SHAP features obtained for the Kitsune dataset.

Model	Kitsune dataset
	SHAP features
Attention-enhanced 1D CNN	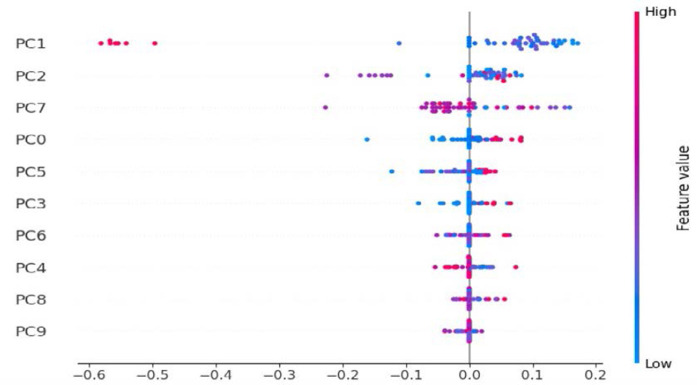
LSTM	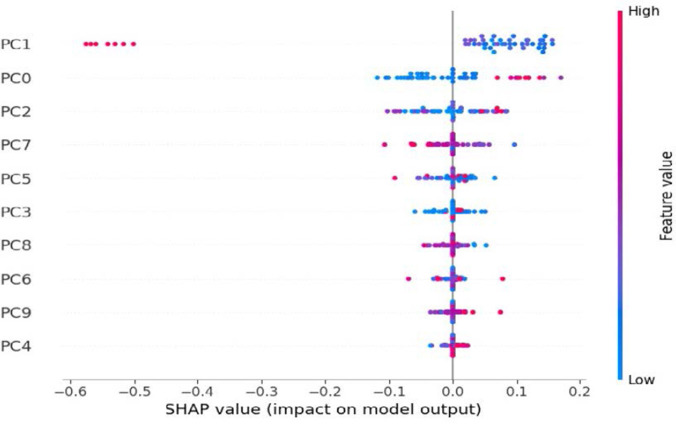
GRU	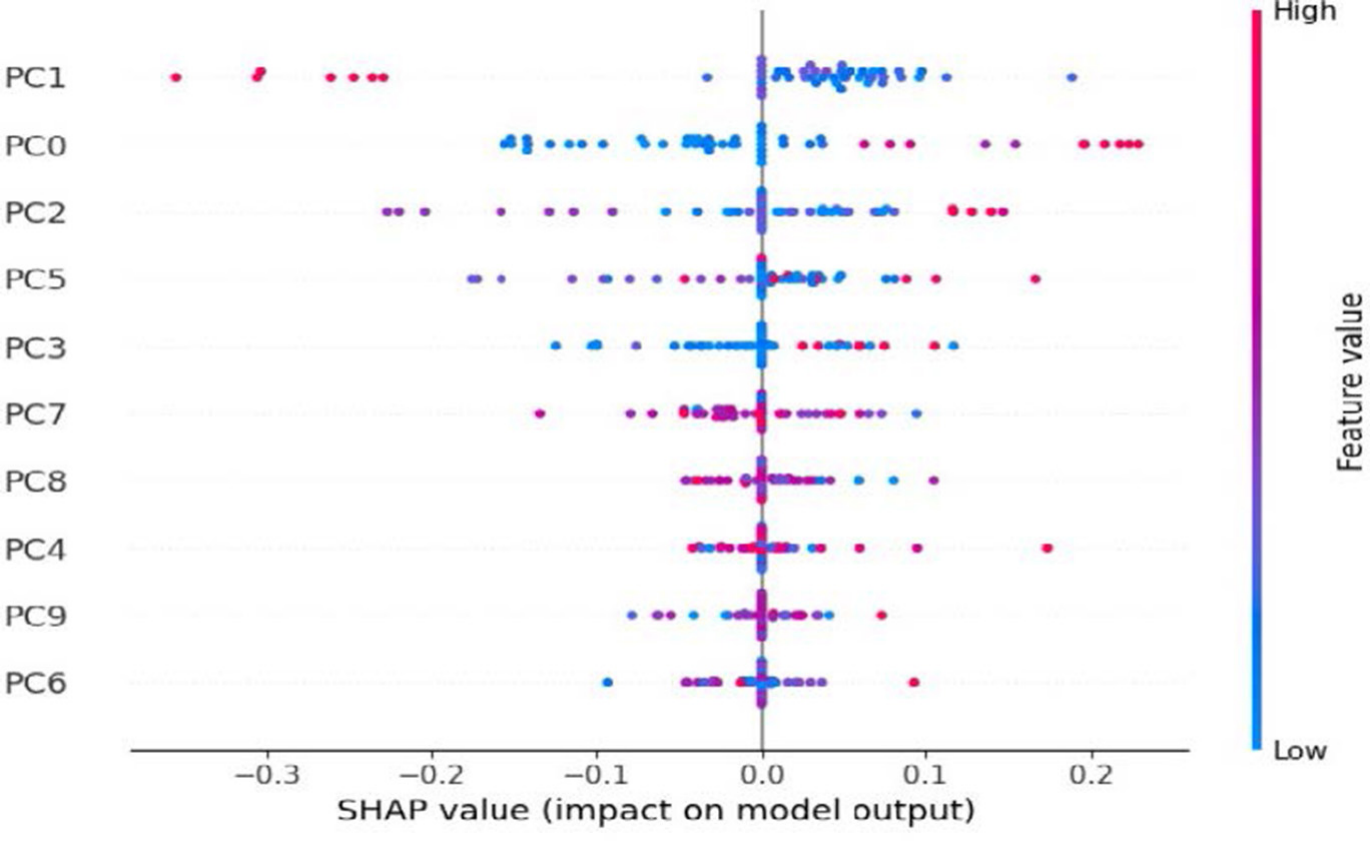

SHapley Additive exPlanations (SHAP) is applied to interpret Attention-enhanced 1D CNN, LSTM, and GRU predictions on the Kitsune Network Intrusion Dataset, as shown in Table 10. Due to the high-dimensionalality of the features, PCA is used to capture the most significant variance. KernelExplainer computed per-class SHAP values using 50 representative training samples, reshaped to match the sequential input format. SHAP summary plots show how each principal component contributes positively or negatively to predicting specific attack types. This approach provides efficient, model-agnostic interpretability, highlighting the most influential patterns in network traffic for normal and malicious behavior.

### 4.8 Performance comparison with existing intrusion-detection models

Table 14 presents a comparative analysis of the accuracy and effectiveness of the proposed architecture, along with LSTM and GRU, for intrusion detection for the IoT HCRL and Kitsune datasets. (Abuzir and Abu Khalil 2025) introduced the Kitsune Fuzzing dataset. They evaluated multiple intrusion-detection methods, including LDA, Logistic Regression and a hybrid CNN–GRU–LSTM model, where Logistic Regression achieved an accuracy of 79% on fuzzing-based attack patterns. Maray et al. (2022) proposed the HSAFS-OCAE framework for SDN-IoT intrusion-detection, integrating Harmony Search Optimization and an optimized Convolutional Autoencoder along with Artificial Fish Swarm Algorithm, achieving an accuracy of 93.44%. Xue et al. (2025) developed a Hybrid Autoencoder–ResNet–LSTM model evaluated on NSL-KDD, UNSW-NB15, and CICIDS-2018, obtaining a high accuracy of 94.9%. Li et al. (2024) introduced a VAE-WGAN combined with LSTM and MSCNN for intrusion detection on NSL-KDD and AWID, attaining an accuracy of 83.45%. Zelichenok and Kotenko (2024) presented a Big Data-driven LSTM-based NIDS for the Kitsune dataset, achieving 93% accuracy, demonstrating the strength of sequential deep learning models in IoT traffic analysis.

**Table 14:** Comparison of proposed model with the state-of-the-art approaches.

References	Dataset/subset	Method	Accuracy (%)	Type
Abuzir and Abu Khalil (2025)	Kitsune Fuzzing	Logistic regression	79	ML
Maray et al. (2022)	SDN IoT	HSAFS-OCAE	93.44	DL/IDS
Xue et al. (2025)	NSL-KDD, UNSW-NB15, CICIDS-2018	Hybrid autoencoder–ResNet-LSTM	94.9	DL
Li et al. (2024)	NSL-KDD, AWID	VAE-WGAN + LSTM + MSCNN	83.45	DL
Zelichenok and Kotenko (2024)	Kitsune	Big Data + LSTM NIDS	93	DL
Proposed	HCRL Kitsune	Attention-enhanced 1D CNN	87 96	DL

## 5 Real-time generalization of a synthetic traffic simulator

To evaluate real-time generalization, a synthetic traffic simulator is constructed to generate 5,000 packet streams in batches of 100 with three scenarios: normal (30% attack ratio), under attack (60% attack ratio), and concept drift (40% attack ratio with shifted feature distributions). The models generated accuracy values, ranging 0.14–0.36, showing the synthetic traffic distributions deviate sufficiently from the training distribution to prevent meaningful discrimination between Benign, DoS, and Mirai classes for the HCRL dataset. Inference latency ranged 64–80 ms per batch, indicating better computational performance for deployment in real-time IDS pipelines. Table 15 shows the performance metrics like balanced accuracy, average latency, and MCC simulated results for HCRL data, and Table 16 shows the Streaming Accuracy for HCRL data.

**Table 15:** Performance Comparison of simulated results for the HCRL dataset.

HCRL dataset	Normal		Under attack		Concept drift	
	Balanced accuracy (%)	Average latency (ms)	Balanced accuracy (%)	Average latency (ms)	Balanced accuracy (%)	Average latency (ms)
Attention-enhanced 1D CNN	0.19	79.54	0.32	64.21	0.23	70.10
LSTM	0.14	71.98	0.25	67.64	0.18	72.70
GRU	0.18	71.87	0.36	71.67	0.24	72.91

**Table 16:** Streaming accuracy for HCRL dataset.

Model scenario	Streaming accuracy (mean batch accuracy)
Attention-enhanced 1D CNN	
Normal	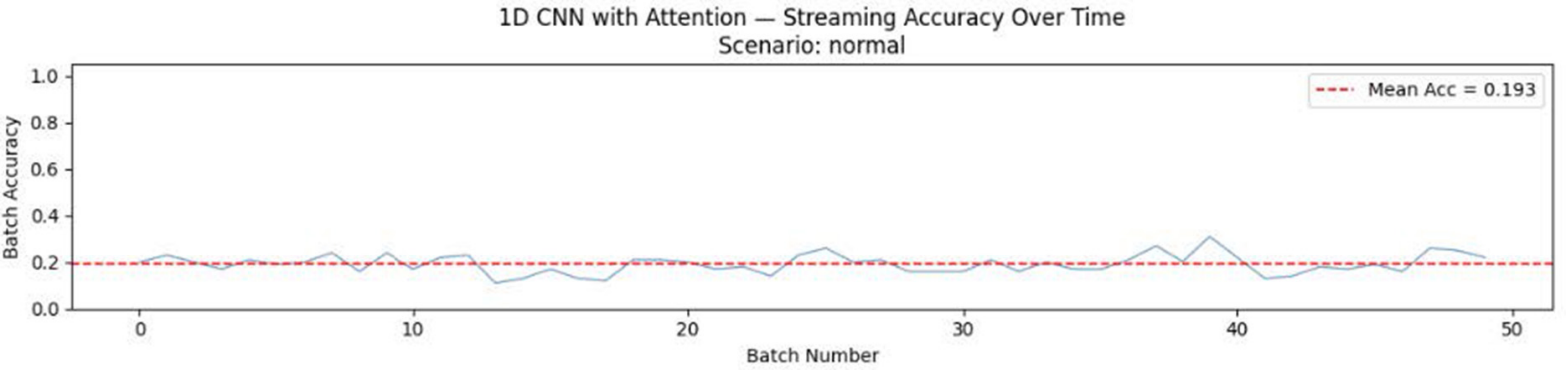
Under attack (attack ratio = 60%)	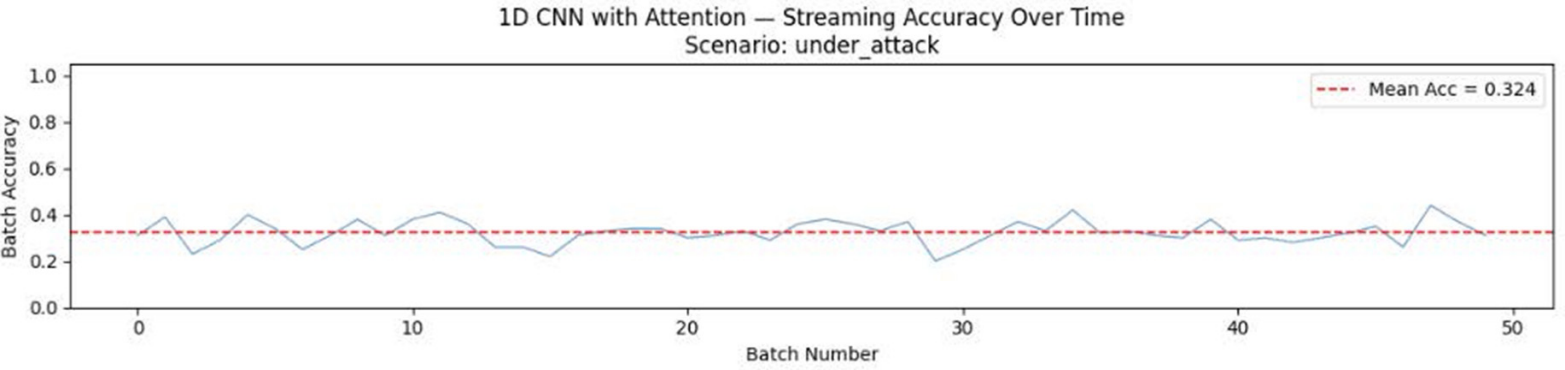
Concept drift (attack ratio = 40%)	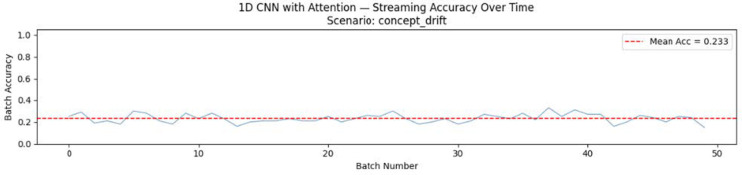
LSTM	
Normal	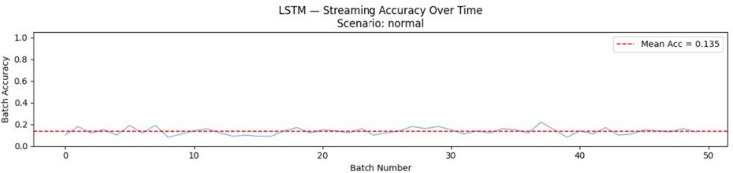
Under attack (attack ratio = 60%)	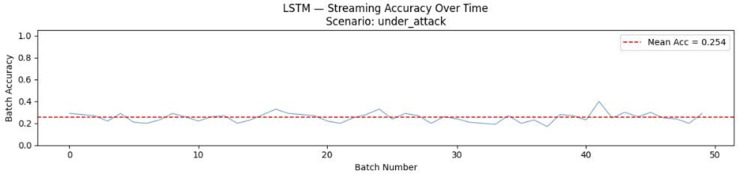
Concept drift (attack ratio = 40%)	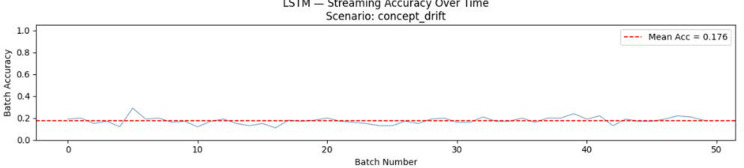
GRU	
Normal	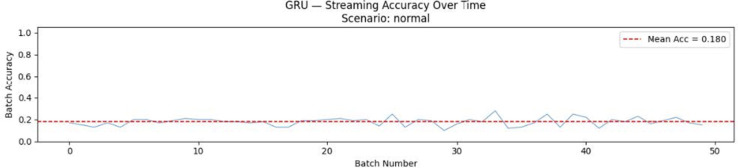
Under attack (attack ratio = 60%)	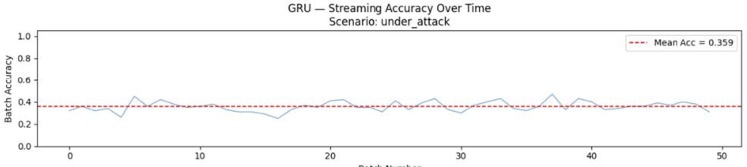
Concept drift (attack ratio = 40%)	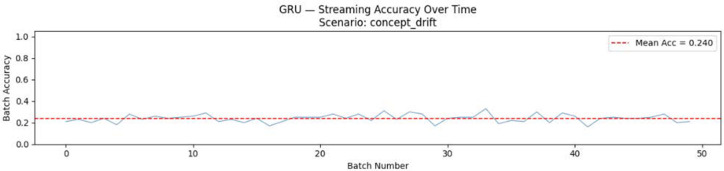

Inference latency was measured using 100-packet streaming batches across a 5,000-packet simulation. The attention-enhanced 1D CNN showed the lowest and most consistent latency with a mean of 68.64 ms, ranging 65.43–75.07 ms per batch. The LSTM attained a mean latency of 72.04 ms, with a spread range of 68.40–79.62 ms, while the GRU achieved a mean of 72.15 ms, with a slightly wider range extending to 94.44 ms, indicating occasional processing spikes under streaming conditions. All three models processed each 100-packet batch within 100 ms, demonstrating near-real-time intrusion detection with low-latency inference.

**Figure 7:**
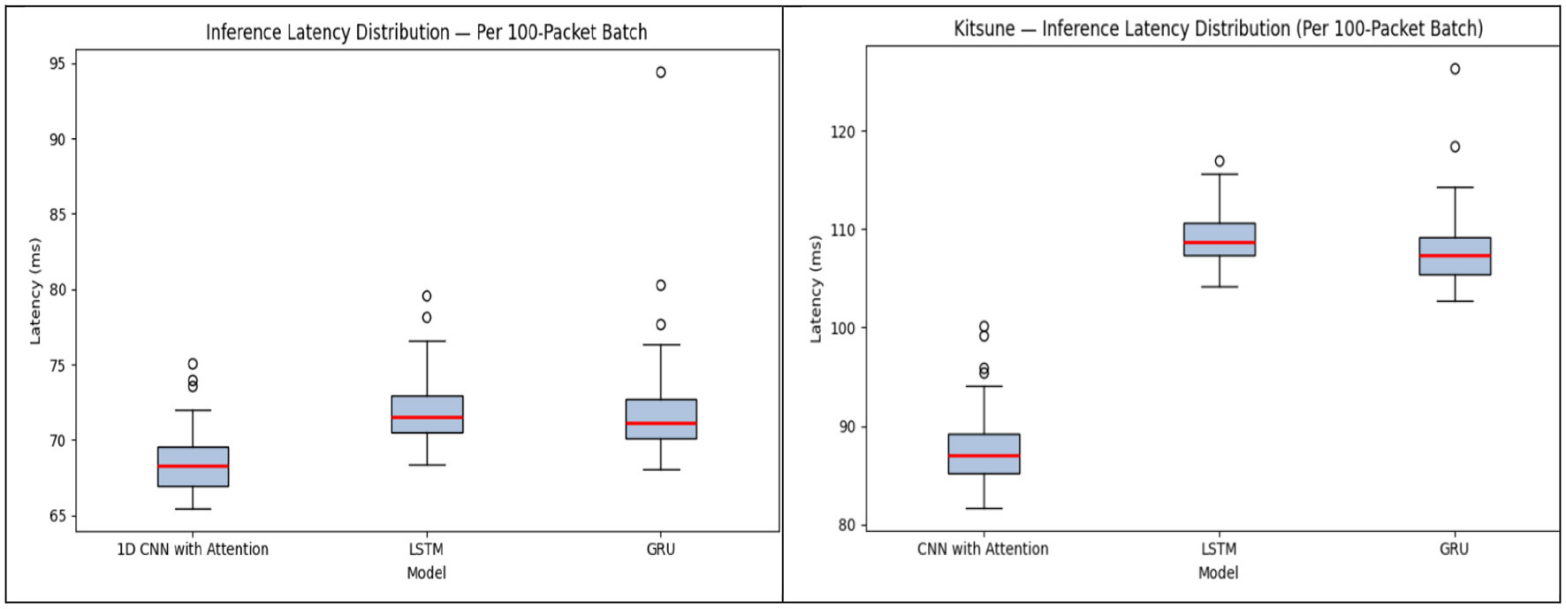
Latency distribution with per 100 packet batch for the Kitsune dataset.

Kitsune dataset (Tables 17, 18) attained 0.70 under the Normal scenario for the LSTM and GRU, while the attention-enhanced 1D CNN reached 0.69, showing strong generalization. Performance dropped to 0.40 for all models under the under attack scenario, while the concept drift scenario yielded a partial recovery at 0.59–0.60, indicating moderate robustness to distribution shifts. Inference latency ranged from 84.56 ms to 123.23 ms per batch across all models and scenarios, with every model processing each 100-packet batch within 125 ms, confirming suitability for near-real-time IDS deployment. Figure 7 shows the Inference latency for data streaming batches of 100 packets over a 5,000-packet simulation.

**Table 17:** Performance comparison of simulated results for the Kitsune dataset.

Kitsune dataset	Normal		Under attack		Concept drift	
	Accuracy (%)	Average latency (ms)	Accuracy (%)	Average latency (ms)	Accuracy (%)	Average latency (ms)
1D attention enhanced CNN	0.69	100.63	0.40	84.56	0.59	89.92
LSTM	0.70	111.27	0.40	123.23	0.60	113.26
GRU	0.70	110.43	0.40	112.04	0.60	113.30

**Table 18:** Streaming accuracy for Kitsune dataset.

Model scenario	Streaming accuracy (mean batch accuracy)
Attention-enhanced 1D CNN	
Normal	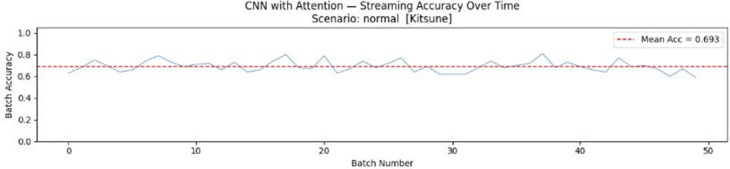
Under attack (attack ratio = 60%)	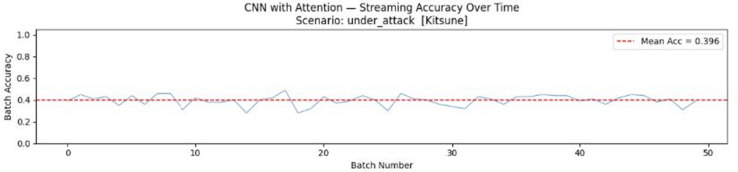
Concept drift (attack ratio = 40%)	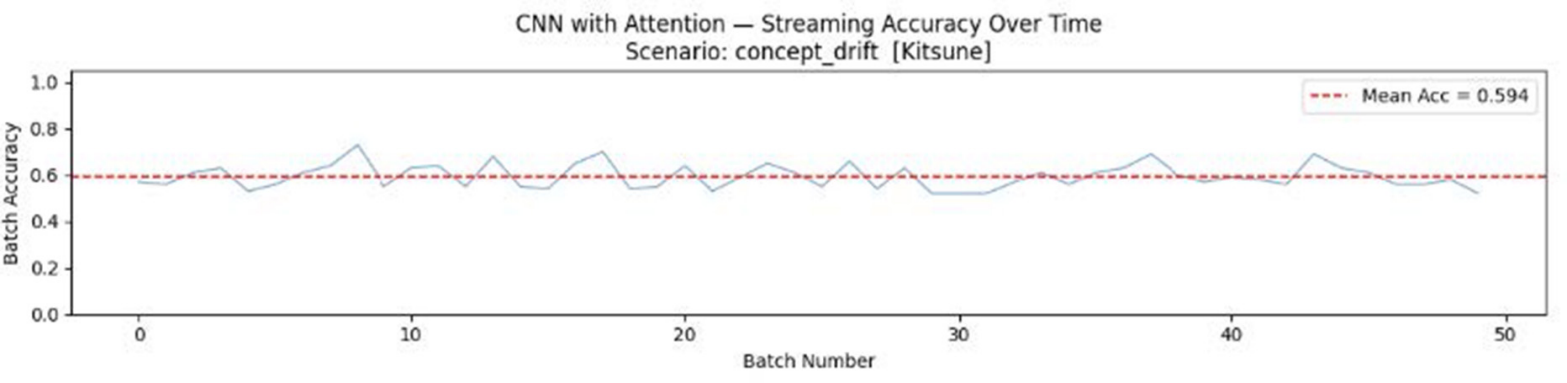
LSTM	
Normal	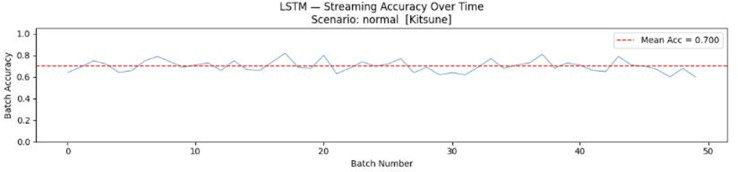
Under attack (attack ratio = 60%)	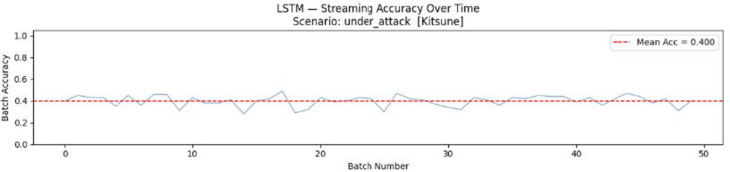
Concept drift (attack ratio = 40%)	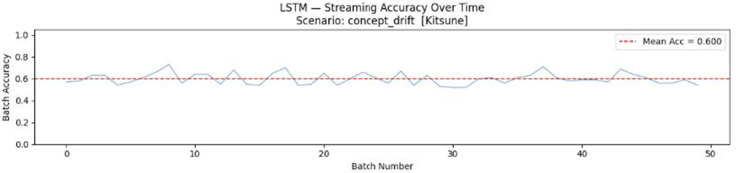
GRU	
Normal	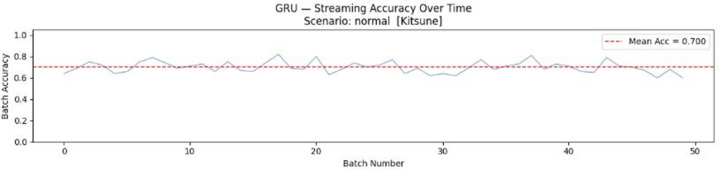
Under attack (attack ratio = 60%)	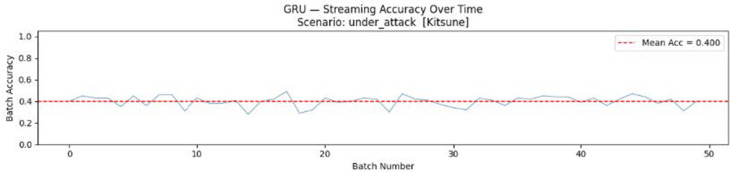
Concept drift (attack ratio = 40%)	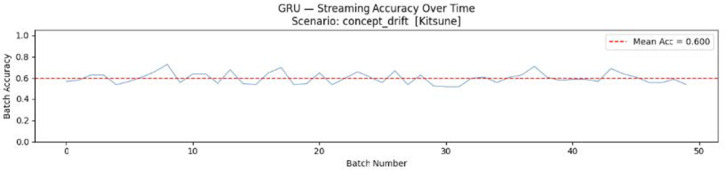

## 6 Conclusion and future work

This study presents a comprehensive intrusion-detection framework attention-enhanced 1D CNN, along with LSTM and GRU architectures for robust multi-class attack detection in IoT and network environments. The proposed attention-enhanced 1D CNN model enhances feature extraction by combining convolutional layers with channel and spatial attention modules, enabling the network to selectively emphasize the most informative temporal and statistical attributes of the input traffic. LSTM and GRU models effectively captured sequential dependencies and temporal correlations in network flows, resulting in strong discrimination across various attack categories. SHAP-based explainability further strengthens the interpretability of the system by highlighting feature contributions for each class providing valuable insights for cybersecurity analysts. Experimental results confirm the effectiveness of the proposed models, where the attention-enhanced 1D CNN achieves superior performance compared to recurrent models, reflected by its classification accuracy as 96% for Kitsune and 87% for the HCRL dataset, and consistent generalization across test samples. The proposed work contributes an interpretable, efficient, and scalable deep learning model for IDS suitable for real-world IoT and network security applications. Future work should focus on extending the models to handle larger and more diverse real-world network environments, including encrypted and IoT-specific traffic. Integrating transformer or graph learning architectures could further enhance long-range dependency detection and improve robustness against emerging attack patterns. Improving class balance by incorporating synthetic data generation or integrating enhanced attention mechanisms could help strengthen the detection of minority classes in future work.

## Data Availability

The original contributions presented in the study are included in the article/supplementary material, further inquiries can be directed to the corresponding author.
